# *Nicotiana glauca* leaf extract exhibits synergistic antioxidant and anticancer activities revealed by phytochemical profiling and molecular docking

**DOI:** 10.1038/s41598-026-67585-9

**Published:** 2026-09-03

**Authors:** Marwa M. H. Saeed, Sayed A. Fayed, Rim Hamdy, Emad A. Shalaby

**Affiliations:** 1 Biochemistry Department, Faculty of Agriculture, Cairo University, Giza, 12613 Egypt; 2 Botany and Microbiology Department, Faculty of Science, Cairo University, Giza, 12613 Egypt; 3 Biological Sciences Department, Faculty of Science, Galala University, Suez, Egypt

**Keywords:** *Nicotiana glauca*, Wild plants, Antioxidant activity, Combination index, Molecular docking, Biochemistry, Biotechnology, Cancer, Chemical biology, Chemistry, Drug discovery, Plant sciences

## Abstract

This study aimed to characterize the phytochemical composition and antioxidant, cytotoxic and molecular docking profile of *Nicotiana glauca* Graham (Solanaceae), a wild plant species naturalized along the Wadi El Natrun–North Coast Road, Egypt. *N. glauca* was selected from a preliminary screening of ten co-occurring wild plant species collected during the summer of 2022 on the basis of its highest extraction yield (2.20%), the richest qualitative phytochemical profile among the ten species, and its strong antioxidant activity. The aqueous ethanolic leaf extract of *N. glauca* and its sixteen column fractions were subsequently evaluated using four chemical antioxidant assays: 2,2-diphenyl-1-picrylhydrazyl (DPPH), 2,2′-azinobis(3-ethylbenzothiazoline-6-sulfonic acid) (ABTS), potassium permanganate (KMnO₄), and dichlorophenolindophenol (DCPIP). Comprehensive phytochemical characterization was performed using thin-layer chromatography (TLC), TLC-bioautography, Fourier-transform infrared spectroscopy (FTIR), mass spectrometry, ^1^H and ^13^C nuclear magnetic resonance (NMR), and GC/MS/MS analyses. Total phenolic, flavonoid and alkaloid contents ranged from 0.3–8.4 g GAE/100 g DW, 0.1–2.14 g/100 g DW, and 0.31–7.01 g/100 g DW, respectively, across the extracts; a Pearson correlation analysis between these compound classes and antioxidant activity across the ten species did not reach statistical significance (p > 0.05), consistent with a ceiling effect at the tested concentration and supporting a combined rather than single-compound-class contribution to activity. GC/MS/MS analysis of fraction 11 revealed anabasine as the predominant alkaloid (39.26%), followed by nornicotine (36.50%). Two pure compounds were isolated and identified as N,N-methylenebis(14-docosene) (Compound A) and 14-methylhexadecanoic acid (Compound B). A synergistic interaction was observed between the crude extract and Compound A for both antioxidant (combination index, CI = 0.53) and anticancer activity (CI = 0.139–0.158), whereas an antagonistic effect was observed with Compound B (CI = 2.2–28.8). Both the crude extract and isolated compounds exhibited concentration-dependent cytotoxic activity against HeLa (IC₅₀ = 146.04 µg/mL) and HepG2 (IC₅₀ = 169.38 µg/mL) cancer cell lines. Treatment of HeLa cells with Compound A was associated with upregulation of the pro-apoptotic Bax gene, downregulation of the anti-apoptotic Bcl-2 gene, an increased Bax/Bcl-2 ratio, and elevated malondialdehyde (MDA) levels, findings consistent with, though not direct proof of, apoptosis induction and oxidative stress. Molecular docking analysis indicated that anabasine binds with favorable predicted affinity to the HPV16 E2 (PDB: 2Q79) and E6 (PDB: 7UAJ) oncoproteins; these computational results provide hypothesis-generating support for further exploration of anabasine against HPV-associated oncoproteins and require experimental validation. Overall, these findings characterize *N. glauca* as a promising wild source of antioxidant and cytotoxic bioactive metabolites and support further mechanistic and translational investigation.

**Supplementary Information:**

The online version contains supplementary material available at https://doi.org/10.1038/s41598-026-67585-9.

## Introduction

Medicinal and aromatic plants represent a vital aspect of biodiversity, contributing significantly to both healthcare and the economy; historically, these plants have been employed for diverse therapeutic purposes, and numerous bioactive compounds with pharmacological potential have been isolated from plant sources, particularly in the Middle East^1,2^.

Drug discovery remains a key global priority, and growing concerns over the side effects of synthetic drugs have renewed interest in natural products, including herbs, spices, and plant extracts, whose complex and variable chemical composition is shaped by environmental and processing factors^3,4^.

Wild plants remain a primary source of diverse secondary metabolites — including phenolic compounds, organic acids, and terpenoids — with antimicrobial, anti-inflammatory, antioxidant, and anticancer activities^5,6^. Globally, oxidative stress underlies much of the burden of acute and chronic disease, and plant constituents capable of neutralizing reactive oxygen species are of considerable therapeutic interest^7^; aromatic-plant bioactives more broadly support their traditional use against various ailments^8,9^, with antioxidant, antiviral, antimicrobial, and anticancer effects of essential oils largely attributed to terpenoid compounds^10–12^. Cancer remains a major global health, societal, and economic burden^13^, and despite advances in conventional therapy, drug resistance and severe side effects continue to drive interest in novel, plant-derived anticancer agents. A key challenge in exploiting such complex extracts is that interactions among their constituents — quantifiable through the Combination Index (CI) framework — can be synergistic or antagonistic and are therefore critical to therapeutic optimization^14^; in parallel, molecular docking serves as a predictive tool for exploring binding affinities between plant-derived compounds and target proteins, offering insight into potential mechanisms of action^15^.

*Nicotiana glauca* Graham (Solanaceae), commonly known as tree tobacco, is native to South America and widely naturalized as an invasive species across arid and semi-arid regions of the Mediterranean basin, including the Egyptian North Coast and Wadi El-Natrun depression, where it colonizes disturbed, saline, and roadside habitats. Botanically, it is a glabrescent shrub or small tree 2–6 m tall; the petiole is slender, 3–12 cm long, and the leaf blade is ovate, 5–25 cm, leathery, glaucous, with an obtuse-to-cordate base, an entire margin, and an obtuse or acute apex. The inflorescence is a many-flowered, lax panicle borne on pedicels 3–12 mm long; the calyx is tubular, 1–1.5 cm, with equal, deltate, acute lobes, and the corolla is yellow to red, tubular, 2.5–4.5 cm, with short lobes and subequal, included stamens. The fruit is an ellipsoid capsule, 0.7–1.5 cm, containing numerous brown seeds approximately 0.5 mm long. The species accumulates piperidine alkaloids — principally anabasine, with nornicotine and nicotine as minor constituents — as a chemical defense strategy, distinguishing its alkaloid profile from the nicotine-dominant profile of cultivated *Nicotiana tabacum*L^16–18^..

Prior phytochemical work on *N. glauca* has reported notable phenolic and flavonoid content together with antioxidant and anti-inflammatory activity in methanolic leaf extracts^19^, antibacterial and antioxidant properties associated with related Nicotiana stem extracts^20^, and anthelmintic/anticancer screening of crude water extracts against MCF-7 and HeLa cells^21,22^. GC–MS surveys of *N. glauca* have consistently identified anabasine as its predominant alkaloid^23^, and its toxicological potential, including a documented case of fatal human poisoning, is well established^24^. However, to our knowledge, no previous study has combined (i) a comparative ten-species wild-plant screening used explicitly to select *N. glauca* on quantitative grounds, (ii) bioassay-guided fractionation down to two structurally characterized pure compounds, (iii) Combination-Index-based quantification of synergy/antagonism between the crude extract and each isolated compound for both antioxidant and anticancer endpoints, (iv) apoptosis-related gene expression and oxidative-stress biomarker analysis for the most synergistic compound, and (v) molecular docking of the predominant alkaloid, anabasine, against HPV oncoprotein targets. This integrated screening-to-mechanism pipeline, and in particular the synergy quantification between the crude extract and its isolated constituents, represents the principal novelty of the present work relative to previous *N. glauca* studies.

The present study therefore aims to: **(i)** identify and justify *Nicotiana glauca* as the most promising candidate emerging from a comparative screening of ten wild-plant species collected from the Egyptian North Coast; **(ii)** comprehensively characterize the phytochemical composition of the bioactive *N. glauca* fractions and isolate their principal constituents; **(iii)** quantitatively assess the interactions, including synergy and antagonism, between the crude extract and its isolated compounds in antioxidant and anticancer assays using the Combination Index (CI) method; **(iv)** investigate the apoptotic and oxidative-stress-related mechanisms underlying the activity of the most synergistically interacting compound in HeLa cells; and (v) explore, as a hypothesis-generating computational approach, the potential binding interactions of the predominant alkaloid, anabasine, with high-risk human papillomavirus (HPV) oncoprotein targets.

Importantly, the novelty of the present study lies in its integrated experimental strategy, linking comparative wild-plant screening and phytochemical characterization with quantitative interaction analysis between the crude *N. glauca* extract and its isolated constituents, followed by mechanistic investigation of the most synergistic compound and exploratory molecular docking against high-risk HPV targets. This integrated approach provides a more comprehensive assessment of N. glauca leaf extract and its constituents than evaluating their antioxidant or anticancer activities individually.

## Materials and methods

### Chemicals and reagents

Pure *n*-hexane, chloroform, ethyl acetate, ethanol, and methanol were purchased from E. Merck Co. (Darmstadt, Germany). Sigma-Aldrich (St. Louis, MO, USA) supplied sulforhodamine B (SRB), 2,2-diphenyl-1-picrylhydrazyl (DPPH), and 2,2'-azino-bis (ethylbenzothiazoline-6-sulfonic acid) (ABTS). Sigma-Aldrich also provided butylated hydroxytoluene (BHT) and gallic acid. Doxorubicin (DOX; Sigma-Aldrich, St. Louis, MO, USA) was used as a reference anticancer standard for the cytotoxicity assays.

### Collection of plant materials

Fresh wild specimens were collected in August 2023 from the Wadi El Natrun region along the Alexandria Road, Egypt. The sampling area spans a latitudinal range of 30°30′N to 30°54′N (north–south direction) and a longitudinal range of approximately 10′E to 50′E (east–west direction).

**Fig. 1:**
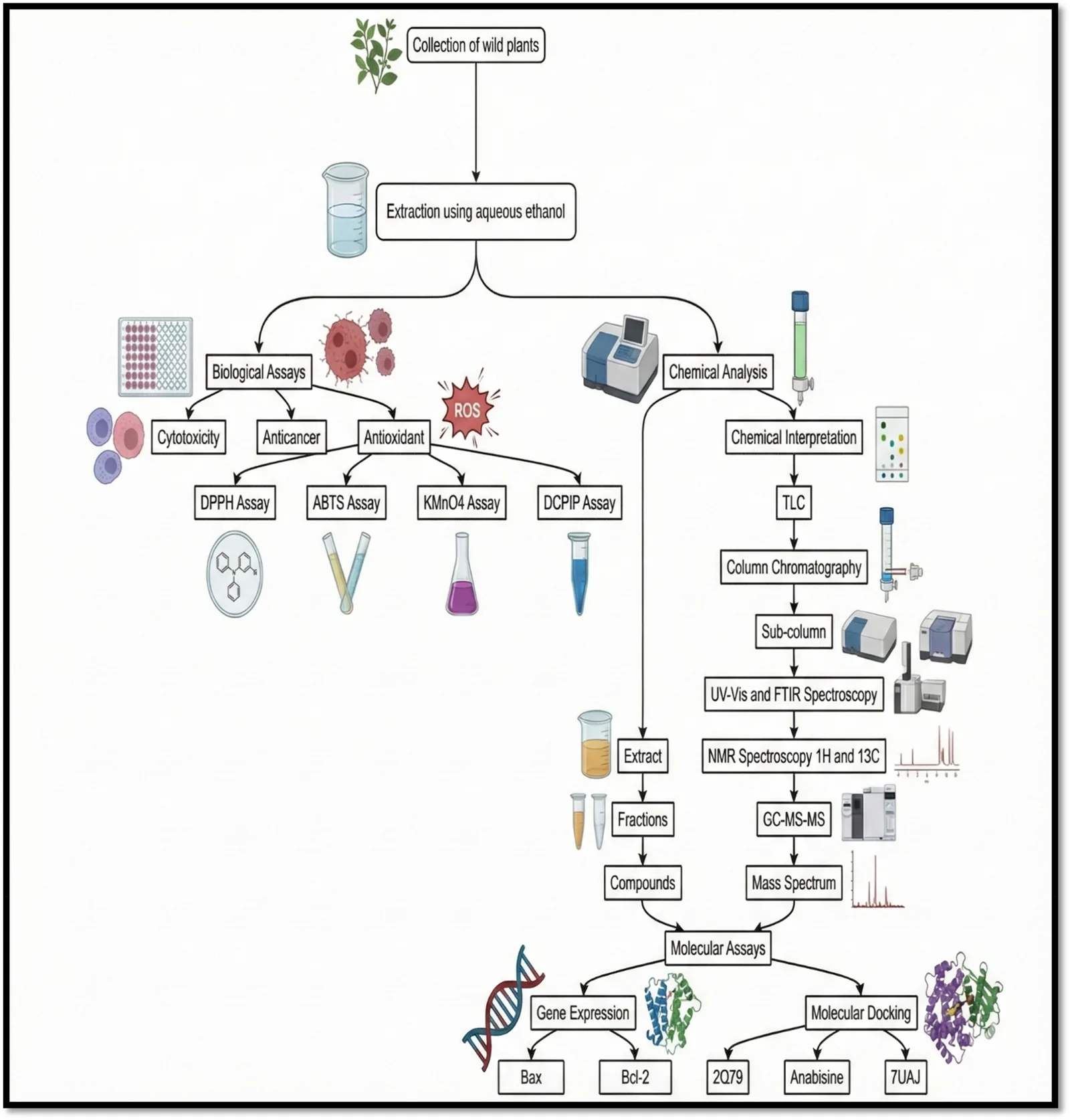
Schematic workflow of the experimental design and analysis of wild plants.

The formal identification and taxonomic verification were conducted by Rim Hamdy, Professor of Taxonomy and Flora in the Department of Botany and Microbiology, Faculty of Science, Cairo University, and a member of the Cairo University Herbarium. Identification was compared with authenticated specimens housed at the Cairo University Herbarium (CAI), utilizing diagnostic keys from the local flora. The collected plants were extracted, and the biological and chemical parameters were determined as shown in Fig. 1.

### The specimen vouchers are the following

*Cornulaca monacantha* (CAI 24.89.253.147); *Stipagrostis plumosa* Munro ex T.Anderson (CAI 123.659.1915.251); *Artemisia monosperma* Delile (CAI 105.546.1582.176); *Zygophyllum arabicum* (L.) Christenh. & Byng (CAI 49.246.748.150); *Thymelaea hirsuta* (L.) Endl. (CAI 67.292.922.183); *Bassia indica* (Wight) A.J.Scott (CAI 24.75.210.76); *Erigeron bonariensis* L. (CAI 105.503.1492.98); *Nicotiana glauca* Graham (CAI 93.436.1287.55); *Cynanchum acutum* L. (CAI 86.364.1074.109); *Zygophyllum album* L.f. (CAI 49.248.770.308).

### Compliance with regulations for plant collection

All field studies and collection of wild plant material conducted in this study complied with the relevant institutional, national, and international guidelines and legislation governing the collection and use of plant specimens. The collection of plant material was carried out with the appropriate permissions and/or permits from the relevant authorities. The study was conducted in accordance with the IUCN Policy Statement on Research Involving Species at Risk of Extinction and the Convention on International Trade in Endangered Species of Wild Fauna and Flora (CITES).

### Statement on permissions for plant/seed collection from Wadi El-Natrun region

**Fig. 2:**
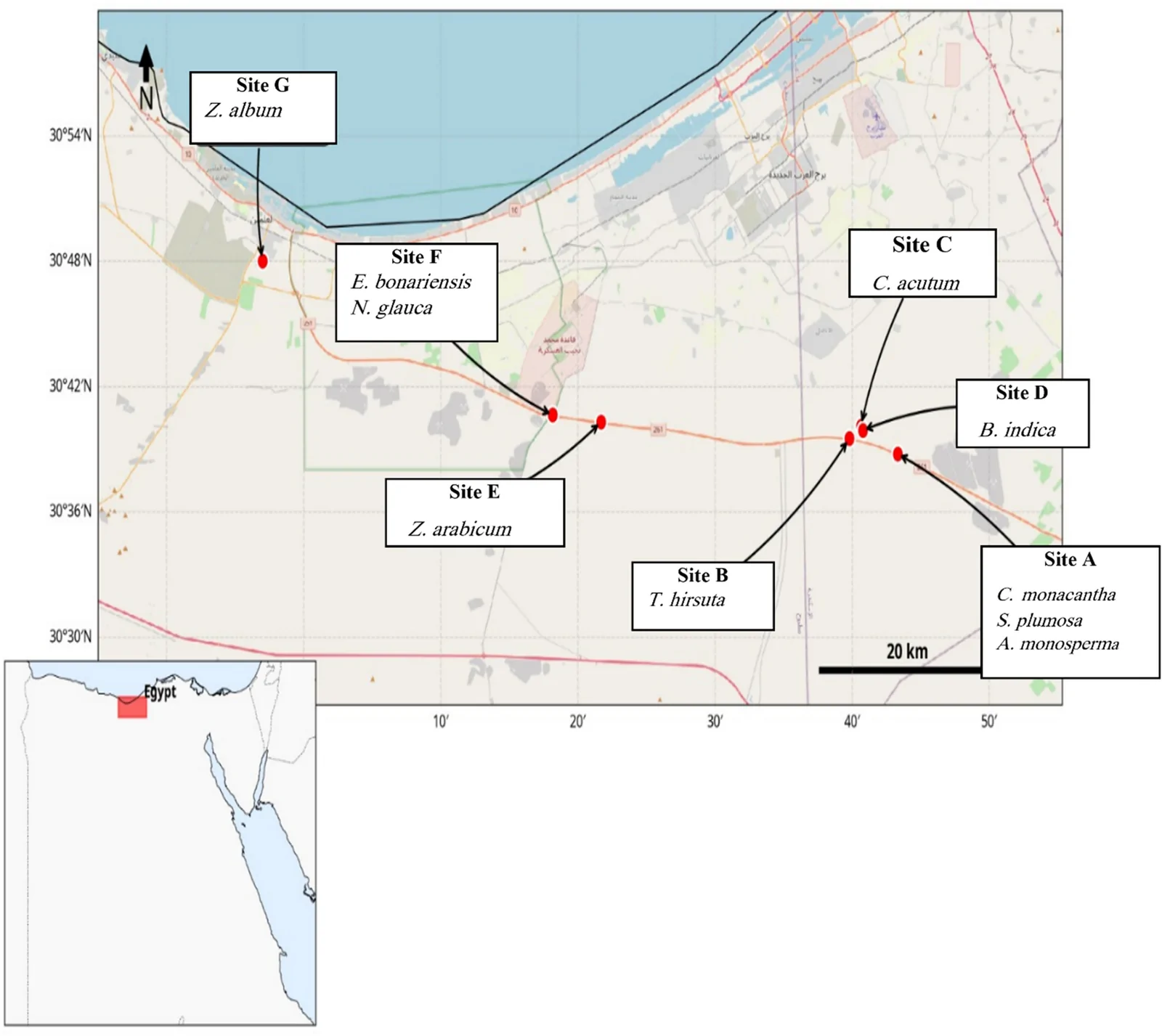
Collection Sites of Selected Wild Plant Species in Northern Egypt.

The plant and seed specimens used in this study were collected from the Wadi El-Natrun region, Alexandria Governorate, Egypt (Fig. 2). This area is a public, non-protected region, and according to local regulations, no special permits or governmental approvals are required for the collection of naturally occurring plant materials for scientific, educational, or non-commercial research purposes.

#### Plant extraction

Air-dried leaves (100 g) were macerated in 600 mL of 70% ethanol for three successive extractions. The combined filtrates were concentrated under reduced pressure and stored in brown glass bottles at 4 °C until further analysis^25^.

#### Phytochemical screening of plant extracts

Preliminary qualitative phytochemical screening was performed on all plant extracts to detect the presence of major bioactive compound classes. The screening included tests for: phenolic compounds, flavonoids, proteins, coumarins, alkaloids, glycosides, and/or carbohydrates according to the methods described by^25,26^.

Unless otherwise stated, all biological assays described in this section (antioxidant assays, cytotoxicity/MTT assays, gene expression analysis, and MDA determination) were performed with n = 3 independent biological replicates, defined here as three separate experiments performed on three different days, each using an independently prepared extract/fraction dilution series and, for cell-based assays, cells from a different passage; this is distinct from technical replicates (repeated wells within the same run), which were averaged within each biological replicate prior to statistical analysis. The corresponding n is additionally stated in each figure and table legend.

## Determination of antioxidant activity of wild plant extracts using different antioxidant methods (radical and non-radical assays)

### DPPH radical scavenging activity

The antioxidant activity of aqueous ethanolic extracts from different wild plants was evaluated following the method described in^27^.

### ABTS radical cation scavenging assay

This assay was predicated on the capacity of diverse substances to scavenge the 2, 2’-azino-bis (ethylbenzthiazoline-6-sulfonic acid (ABTS. +)) radical cation, with a comparison made against a BHT standard^28^.

### KMnO_4_ as a non-radical scavenging activity

The scavenging effects of aqueous ethanolic extracts from wild plants were ascertained as described in^29^.

### DCPIP scavenging assay

The scavenging effects of aqueous ethanolic extracts were determined through the following protocol: 1.0 mL of a 0.05 g/100 mL DCPIP solution (soluble in 0.04% sodium bicarbonate) was introduced into a test tube containing a 1.0 mL aliquot of the sample (at various concentrations). After a minute of vortexing, the mixture was left to stand at room temperature for five minutes in the dark. The absorbance of all sample solutions was measured at 600 nm using a spectrophotometer. The percentage of inhibition activity was calculated as follows: % Inhibition = [(AbsC—AbsE)/AbsC] × 100, where 'AbsC' signifies the absorbance of the 0.05% DCPIP solution, and 'AbsE' represents the extract absorbance.

## Determination of phytochemical compounds

### Determination of total phenolic compounds

Total phenolic compounds were quantified utilizing the method detailed in^30^. The phenolic content was quantified from a gallic acid standard curve.

### Determination of total flavonoids

Flavonoid content was determined following the method described in^31^. The quercetin was used as the standard to construct the calibration curve.

## Thin-layer chromatography (TLC) bio-autography for antioxidant activity

A rapid TLC screening method for assessing antioxidant activity was employed, utilizing 2,2-diphenyl-1-picrylhydrazyl radical (DPPH) as a spray reagent. TLC was conducted on all wild plant extracts, in addition to a semi-purified fraction of sample No. 8 of *N. glauca*, as previously described by^32^. Antioxidant activity was assessed by spraying the plates with 0.2% DPPH after drying. The appearance of yellow or white spots against a purple background indicated the presence of antioxidant compounds.

## Fractionation of a promising extract of N. glauca using silica gel column chromatography

The chromatographic column (40 cm in length, 2.5 cm in diameter) was prepared by packing with 150 g of silica gel (60–120 mesh for column chromatography), using the dried packing method. A 10 g quantity of the crude ethanolic extract of *N. glauca* (selected based on its promising biological activities) was thoroughly ground with silica gel powder and subsequently loaded onto the top of the packed column. The column was then sequentially eluted with 100% hexane, followed by ethyl acetate, acetone, and methanol solvents. The polarity was incrementally increased by 15% between each mobile phase mixture, resulting in a total of 16 fractions.

## Sub-column of Fractionation of promising plant extract (sample no. 8) using column chromatography

The chromatographic column (40 cm length, 2.5 cm diameter) was packed with 150 g silica gel (60–120 mesh for column chromatography). A total of 5.0 g of Fractionation No. 11 (as a promising fraction) was ground very well with silica gel powder and then placed on top of the packed column. The column was then sequentially eluted with 100% hexane, followed by ethyl acetate and acetone solvents, the polarity increased by 15% between each mobile phase mixture (a total of 7 fractions were obtained.

## Antioxidant activity of semi-purified fractions

Semi-purified fractionation was exclusively performed on the promising *N. glauca* ethanolic extract. A total of 16 fractions, in addition to 7 sub-fractions, were obtained and subsequently evaluated for their antioxidant activity against DPPH and ABTS radicals, as previously described in the radical scavenging assays.

## Determination of combination index (CI) for antioxidant activity

The interaction between the crude *N. glauca* extract and two isolated pure compounds (A and B) was assessed for their antioxidant activity using the Combination Index (CI) method. Adhering to established criteria^33^, a CI value below 0.8 indicates synergism, a value between 0.8 and 1.2 suggests an additive effect, and a value exceeding 1.2 signifies antagonism.

The crude extract and each isolated compound (A or B) were combined at a constant, non-fixed molar/mass ratio spanning a concentration range bracketing each component's individually determined IC₅₀ (or activity-effective concentration), following the median-effect principle of Chou and Talalay^33^. Dose–effect curves for the extract alone, the compound alone, and the combination were fitted to the median-effect equation, and the Combination Index at the relevant effect level was calculated as CI = (D)₁/(Dx)₁ + (D)₂/(Dx)₂, where (D)₁ and (D)₂ are the concentrations of the extract and compound, respectively, required to produce the same effect in combination, and (Dx)₁ and (Dx)₂ are the concentrations required to produce that same effect when each is used alone. Calculations were performed using CompuSyn software (ComboSyn Inc.), consistent with the classification of CI < 0.8 as synergistic, 0.8–1.2 as additive, and CI > 1.2 as antagonistic^33^.

## Phytochemical identification using GC/MS/MS

The chemical constituents of Fraction 11 and its sub-fraction no.7 of the *N. glauca* extract were analyzed using a GC–MS/MS QQQ 7890B GC system (Agilent) equipped with an HP-5MS UI capillary column (30 m × 0.25 mm × 0.25 μm film thickness) with an auto sampler, this column is suitable for analyzing a wide range of samples, including plant extracts and other complex matrices. The GC’s parameters were set up as follows: auxiliary temperature: 280 °C; carrier gas (Helium) flow rate: 1.1 ml/min; inlet temperature: 250 °C; oven temperature: 90 °C at 0 min, increased to 200 °C for 2 min (3 °C/min), then 280 °C for 2 min (15 °C/min). The total retention duration of the chromatographic analysis was 50 min. The following were the MS parameters that were set: quad temperature: 150 °C; source temperature: 230 °C; mass range: 50–650 m/z; mode: scan mode. Compound identification was achieved by comparing their retention times and mass spectra with entries in the WILEY 09 and NIST 11 mass spectral databases. The relative percentage of separated compounds was determined from the peak areas of the total ionic chromatogram (TIC).

## Chemical structure identification of pure isolated compounds

Using pre-coated TLC F254 plates, the separated fractions, which seemed the best according to their biological activities, were scratched and eluted with the same mobile phase, filtered, evaporated, weighed and then used in bioassays. The most potent fractions were chosen for further identification using the chromatographic and spectroscopic methods as follows:

### Scanning UV–vis spectrophotometry

The obtained pure compounds of *N. glauca* were analyzed using UV–Vis spectrophotometry (Thermo Scientific HERYIO γ, Model 9423 UVG-1702 E, Power Supply, UK) to obtain their spectral profiles over 200–800 nm and determine the λ_max values.

### Fourier transform infrared (FTIR) spectrometer analysis

Fourier transform infrared spectroscopy (PerkinElmer FTIR Spectrometer, System 2000, USA) over a scanning range of 400–4000 cm⁻^1^ was used to identify the functional groups of the isolated pure compounds from *N. glauca* leaf extract.

### ^1^H and ^13^C nuclear magnetic resonance (NMR) spectroscopy

^1^H (300 MHz) and ^**1**3^C NMR Spectroscopy were obtained using a Mercury-300 BB NMR spectrometer. The sample for ^1^H analysis was dissolved in deuterated DMSO-d6, and for ^13^C analysis, in deuterated CDCl₃. Standard pulse sequences (zgpul) and processing protocols were employed to optimize resolution and signal-to-noise ratio. The total acquisition time was 58 min and 56 s for 1H and 31 h, 7 min, and 12 s for 13 C analysis.

### Mass spectrometry analysis

Mass spectrometric analysis was performed using an electron ionization (EI) mass spectrometer (Thermo Fisher Scientific). The sample was dissolved in methanol. Key parameters included a Mass Range of 50–700 m/z with an ionizing voltage of 70 eV and a scan time of 1 s per scan. Data acquisition and processing were conducted using Xcalibur software.

## Determination of anticancer activity

### Cell culture and incubation of the tumor cell line

Hela, MCF7 and HepG-2 cell lines were obtained from the Vaccera (Giza, Egypt). Cells were maintained in RPMI-1640 supplemented with 100 µg/mL streptomycin, 100 units/mL penicillin and 10% heat-inactivated fetal bovine serum in a humidified, 5% (v/v) CO2 atmosphere at 37 °C.

### Cytotoxicity assay against cell lines

The cytotoxicity of crude extract and purified fractions of *N*. *glauca* was tested against HeLa, MCF7 and HepG-2 cell lines by MTT assay according to Slater et al.^34^. Doxorubicin was included in each assay as a positive cytotoxic control, tested in parallel with the crude extract and isolated compounds under identical experimental conditions (same cell density, incubation time, and MTT protocol).

To create a full monolayer sheet, 1 × 10^5^ cells/ml (100 μl/well) of cells were added to the 96-well tissue culture plate. The plate was then incubated at 37 °C for 24 h. Following the formation of a confluent sheet of cells, the growth media was decanted from 96-well microtiter plates. With washing media, the cell monolayer was washed twice. The tested material was diluted three times in RPMI medium with 2% serum (maintenance medium), and then the wells were examined with 0.1 ml of each dilution, leaving three as controls and receiving only maintenance media.

The plates were examined after 37 °C incubation. Each well received a 20 µL MTT solution (5 mg/ml in PBS), which was thoroughly mixed with the media by shaking at 150 rpm for 5 min, and the MTT metabolite was incubated at 37 °C with 5% CO2 for 4 h. The media was discarded. (If required, dry the plate on paper towels to remove residue). In DMSO, 200 µL of formazan (the MTT metabolic product) were dispersed. The solvent and formazan were shaken together vigorously for five minutes at 150 rpm. the background was removed, and the optical density was read At 620 nm. Optical density and cell quantity should be directly correlated.

## Determination of combination index (CI) for anticancer activity

The interaction between the crude *N. glauca* extract and two isolated pure chemicals (A and B) was evaluated for their anticancer activities on HeLa and HepG2 cell lines using the Combination Index (CI). Sample interactions were classified as synergistic (CI < 0.8), antagonistic (CI > 1.2), or additive (CI ranging from 0.8 to 1.2)^33^.

## Gene expression analysis

Gene expression analysis was conducted to ascertain the effect of Compound A on the regulation of key apoptotic regulatory proteins in HeLa cells. The expression levels of the pro-apoptotic gene Bax and the anti-apoptotic gene Bcl2 were determined, and the Bax/Bcl2 ratio was calculated to assess the compound's influence on the apoptotic balance. The results were expressed as a fold change compared to untreated control cells. In brief: RNA was extracted from each sample treated cell lines using nuclease-free water and stored at − 70 °C. Concentration and purity of the extracted RNA were determined according to manufacturer’s protocol, RNA was diluted with distilled water and the optical density was measured spectrophotometrically at 260 and 280 nm and the expected absorbance range of pure extracted RNA should be within 1.7 to 2.1 For each sample, extracted RNA (1 µg), random hexamer primer (1 µL) and DEPC-treated water (to 12 1 µL) were mixed, centrifuged briefly and incubated at 65 °C for 5 min. Samples were placed on ice and the following components were added to each sample in the indicated order as recorded in the “QuantiTect Reverse Transcription kit”. The real-time PCR mixture was pre-denatured at 94 °C for 3 min. 35 cycles with each cycle consisting of denaturation at 94 °C for 30 s, followed by extension at 72 °C for 45 s. Reactions were terminated by heating at 72 °C for 5 min. Non-reverse-transcribed RNA controls and no-template negative controls were included to rule out genomic DNA contamination and reagent artifacts. The RT-PCR product at 10 µL was loaded on a 1.5% agarose gel. Band detection was achieved at 100 V for 20–30 min, using a UV transilluminator and visualized under a UV transilluminator after ethidium bromide staining. The PCR product should be visible at 496 bp.

## Measurement of malondialdehyde (MDA) levels

Malondialdehyde (MDA) levels, a well-established biomarker indicative of lipid peroxidation and oxidative damage, were quantified in HeLa cells following treatment with Compound A. Results were presented as fold change relative to the FLD value of the control, with the corresponding IC_50_ value also determined.

The HeLa cell line incubated with pure compound for 48 h can be homogenized on ice in 300 µL of MDA Lysis Buffer (with 3 µL BHT (100X), then centrifuged (13,000 rpm for 10 min) to remove insoluble material. Alternatively, protein can be precipitated by homogenizing a 10 mg sample in 150 µL ddH2O + 3 μl BHT and adding 1 vol of 2 N perchloric acid, vortexing, and centrifuging to remove precipitated protein. Place 200 µL of the supernatant from each sample into a microcentrifuge tube. Add 600 µL of TBA reagent to each vial containing standards and samples. Incubate at 95 °C for 60 min. Cool to room temperature in an ice bath for 10 min. Pipette 200 µL (from each 800 µL reaction mixture) into a 96-well microplate for analysis. Occasionally, samples will exhibit a turbidity that can be eliminated by filtering through a 0.2 µm filter. For colorimetric analysis, read the absorbance at 532 nm.

## Molecular Docking studies (independent computational screening, decoupled from cytotoxicity mechanism)

This docking analysis constitutes a standalone, exploratory computational screen and is explicitly independent of the cytotoxicity findings reported in Section "Determination of anticancer activity" and in the Results; it is not intended, and should not be read, as a mechanistic explanation for the HeLa or HepG2 cytotoxicity results. It is included because anabasine is the predominant alkaloid identified by GC/MS/MS in Fraction 11 (Section "Phytochemical identification using GC/MS/MS"; Table 7) independent of any specific cell line, and the HPV16 E2 (2Q79) and E6 (7UAJ) structures are among the most extensively characterized high-risk HPV oncoprotein crystal structures available in the Protein Data Bank, making them a suitable reference pair for a general, hypothesis-generating assessment of this alkaloid's oncoprotein-binding potential, independent of the HPV genotype associated with the cell lines used elsewhere in this study.

HeLa cells are HPV18-positive, whereas the crystal structures selected for docking (2Q79, 7UAJ) correspond to the HPV16 E2 and E6 oncoproteins, respectively. These targets were therefore selected not as a direct mechanistic explanation of the cytotoxicity observed in HeLa cells, but as representative, structurally well-characterized members of the high-risk HPV oncoprotein family that mediates cervical carcinogenesis more broadly (E6-mediated p53 degradation and E2-mediated regulation of viral oncogene expression). This docking analysis is presented as an exploratory, hypothesis-generating investigation of anabasine's potential anti-HPV-oncoprotein activity, independent of the specific HPV genotype associated with the cell lines used in the cytotoxicity assays.

Molecular docking investigations were performed to elucidate the interaction dynamics of Anabasine with the active sites of two target proteins: 2Q79 (Crystal Structure of single chain E2C from HPV16) and E6 (7UAJ) (Crystal structure of Apo HPV16 E6). These analyses provided critical insights into Anabasine's binding affinity and potential mechanisms of action, thereby laying a foundational basis for further exploration of its therapeutic potential. Then Molecular docking investigations were conducted to explore the dual-targeting potential of 14-Methylhexadecanoic acid and N, N-Methylene-Bis(14-Docosene) against two critical HPV16 viral proteins: the E6 oncoprotein (PDB: 7UAJ)^35^ and the E2 regulatory protein (PDB: 2Q79)^36^. and the HPV18 E6 domain (PDB: 6SJV)^42^. These induced-fit docking simulations aimed to elucidate the binding dynamics and thermodynamic stability of the ligands within the viral active sites, thereby evaluating their efficacy as potential antiviral and anticancer agents.

Protein and ligand structures were prepared using AutoDock Tools (v1.5.6)^37^ and the SwissParam server^38^, respectively. The docking simulations against all three targets (2Q79, 7UAJ, and 6SJV) were executed using AutoDock Vina (v1.1.2)^39^ with an exhaustiveness of 56. To account for induced-fit effects, specific active site residues were treated as flexible during the calculations (Table 1). Post-docking molecular interactions were analyzed and visualized using PyMOL (v3.1.6.1)^40^ and BIOVIA Discovery Studio Visualizer (v25.1)^41^. Additionally, molecular docking against the HPV18 E6 domain (PDB ID: 6SJV) was incorporated into the screening workflow to evaluate comparative binding specificities across high-risk HPV genotypes, with the corresponding scores compiled in Tables 10 and 11.

**Table 1:** Scientific name, family, Arabic name, photos from the origin places and after pressing of the studied plants.

Scientific name	Family	Arabic name	Plant picture from the collected place	Plant picture from the Herbarium process
1. *C. monacantha*	Amaranthaceae	حاذ شوكي/السلي		
2. *S. plumosa*	Poaceae	النصي		
3. *A. monosperma*	Asteraceae	الشيح		
4. Z. *arabicum*	Zygophyllaceae	الشكاعة		
5. *T. hirsuta*	Thymelaeaceae	مثنان أهلب		
6. *B. indica*	Amaranthaceae	القضقاض		
7. *E. bonariensis*	Asteraceae	الأريغارون البوناري		
8.* N. glauca*	Solanaceae	تبغ أزرق		
9.* C. acutum*	Apocynaceae	المديد المدبب/العليق المدبب		
10.* Z. album*	Zygophyllaceae	الرطريط/القِلَّاب/القرمل		

## Statistical analysis

All collected data are presented as the mean ± standard deviation.

Prior to ANOVA, the normality of model residuals was assessed using the Shapiro–Wilk test and the homogeneity of variances was assessed using Levene's test. Because the pooled analysis across all four antioxidant assays showed evidence of heterogeneity of variance and non-normal residuals, the antioxidant activity data were analyzed as four separate two-way ANOVAs (species × concentration), one for each assay (DPPH, ABTS, KMnO₄, DCPIP), each followed by Duncan's multiple range test (DMRT) for post-hoc comparisons. Levene's test confirmed homogeneity of variance for all four assay-specific models (DPPH: p = 0.803; ABTS: p = 0.721; KMnO₄: p = 0.995; DCPIP: p = 0.989). The Shapiro–Wilk test confirmed normally distributed residuals for the DPPH (p = 0.073), KMnO₄ (p = 0.098), and DCPIP (p = 0.680) models. For the ABTS model only, Shapiro–Wilk indicated deviation from normality (p < 0.001), reflecting the very small residual variance characteristic of highly reproducible triplicate measurements, to which the Shapiro–Wilk test is known to be oversensitive at large sample sizes. Two-way ANOVA is well established to be robust to violations of the normality assumption when, as here, the experimental design is balanced (equal replication in every cell) and the homogeneity-of-variance assumption is satisfied^42^; the ANOVA and Duncan's post-hoc results for all four assays are therefore reported with confidence. For each assay, Duncan's Multiple Range Test was applied across all 40 species × concentration combinations (10 species, four concentrations) as a single comparison per assay column; ascorbic acid, included as a reference standard, was excluded from this post-hoc comparison and is reported as mean ± SD only.

For total alkaloid content (Table 3), statistical evaluation across species was performed using One-Way Analysis of Variance (ANOVA) based on dataset summary statistics (mean ± SD, n = 3 independent biological replicates). Where overall statistical significance was established (alkaloid content in plant material, F = 170.88, p < 0.001), mean comparisons were conducted using Duncan's Multiple Range Test at p < 0.05. For variables showing no significant variation across species (alkaloid content in extract, F = 1.26, p = 0.317), post-hoc pairwise comparisons were omitted in accordance with standard statistical practice.

A P-value of less than 0.05 (P < 0.05) was considered statistically significant.

## Results

### Percentage yield of crude plant extracts

**Fig. 3:**
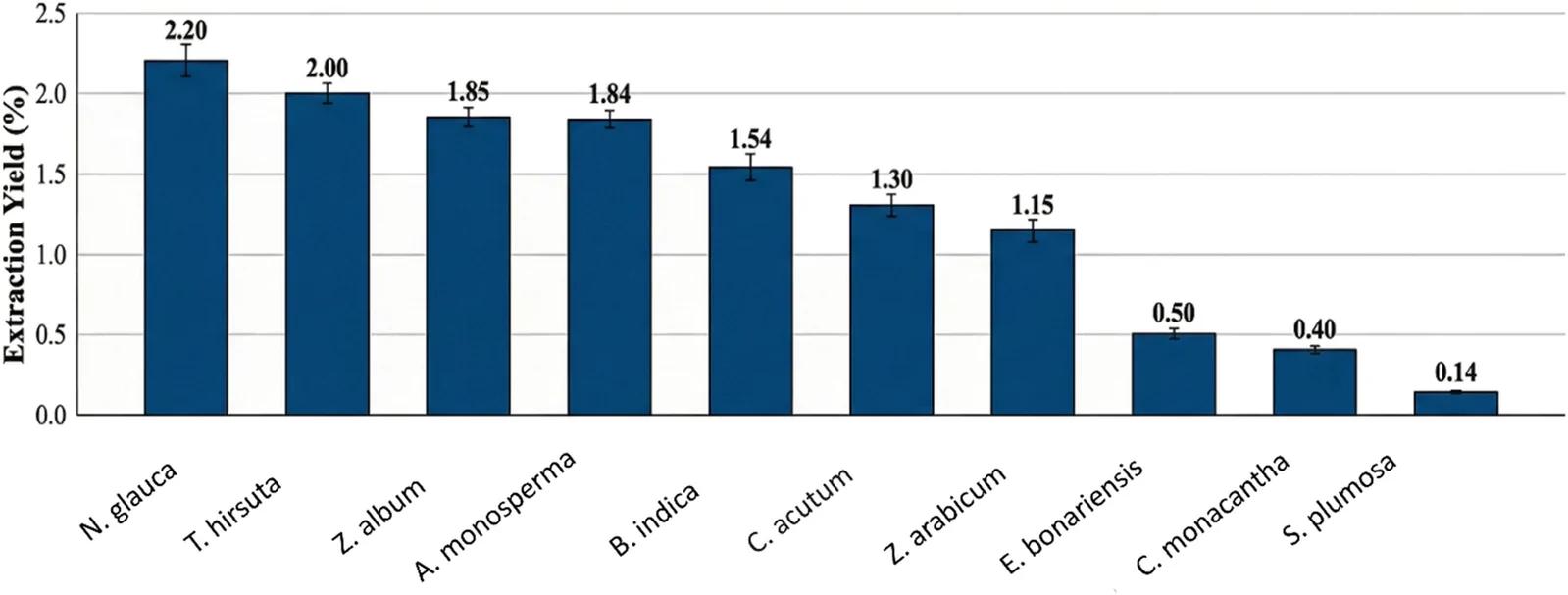
Yield (g/100 g d.wt) of different wild plants collected from the north coast region, Egypt.

The percentage yield of crude extracts obtained from the investigated wild plants ranged from 0.14% to 2.20% (Fig. 3). *N. glauca* exhibited the highest extraction yield (2.20%), followed by *T. hirsuta*. (2.00%), *Z. album* (1.85%), and *A. monosperma* (1.84%).

**Fig. 4:**
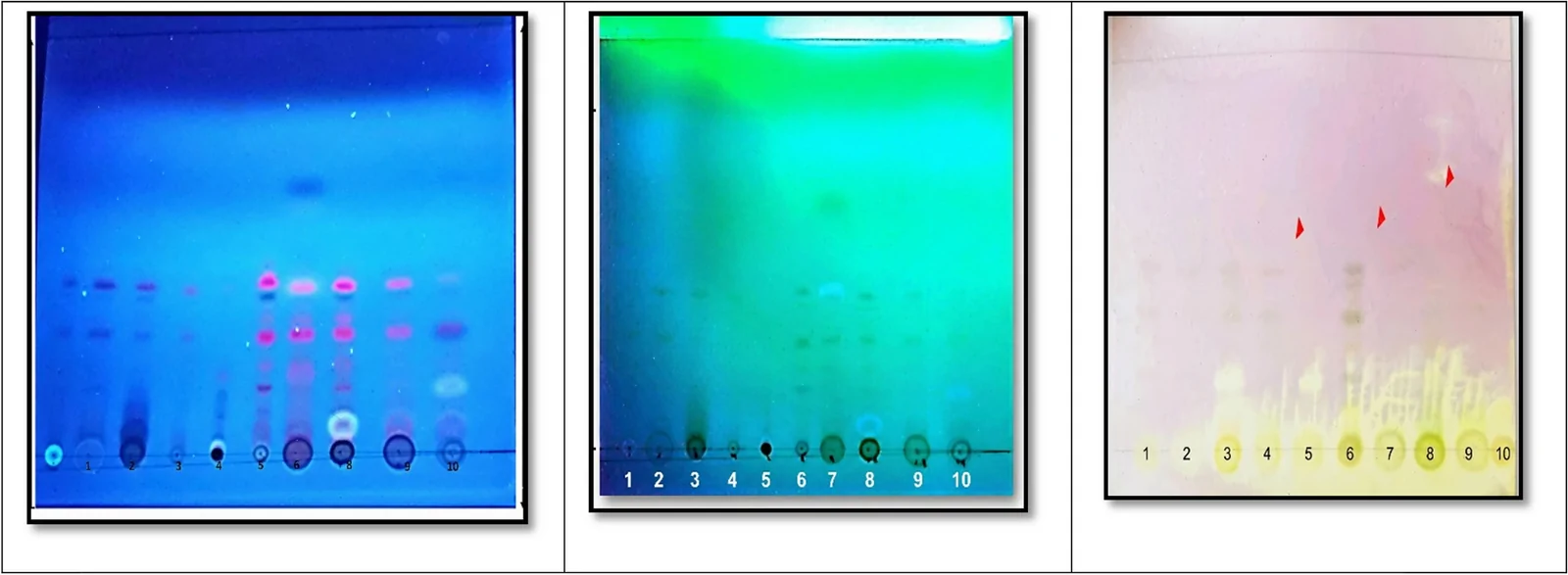
TLC chromatogram (under long and short UV) and TLC bio autography (after sprayed by DPPH reagent) of ethanolic extracts of the wild plants under study (10 plants).

*N. glauca* was prioritized for detailed phytochemical and biological investigation over the other nine screened species on the basis of three converging lines of evidence: (i) it showed the single highest extraction yield among all ten species (2.20%, Fig. 3); (ii) it displayed the richest qualitative phytochemical profile, with the strongest reactions for phenolics, flavonoids, coumarins and tannins of all ten extracts (Table 2); and (iii) it combined near-maximal DPPH and KMnO₄ scavenging activity (Table 5) with the highest alkaloid content recorded in any plant material examined (7.01 g/100 g DW, Table 3) and a strong TLC-bioautography signal (Fig. 4). This combination of high yield, phytochemical richness and biological activity provided the rationale for selecting this species for downstream fractionation, isolation, mechanistic and docking studies.

**Table 2:** Phytochemicals screening of ethanolic extracts from the collected wild plants.

Plant no										
Phytochemical	**1**	**2**	**3**	**4**	**5**	**6**	**7**	**8**	**9**	**10**
Phenolic	** + **	**_**	** + + + **	**_**	** + + **	** + + + **	** + + + **	** + + + + **	** + + **	**-**
Flavonoids	** + **	**_**	** + + + + **	**_**	** + + **	** + **	** + + **	** + + + + **	** + + **	** + **
Protein	** + **	** + **	** + **	** + **	** + **	** + **	** + **	** + + **	** + **	** + **
Coumaric	** + + **	** + + **	** + + + **	** + **	** + + **	** + **	** + **	** + + + + **	** + + **	** + **
Glycoside	** + + **	** + + **	** + + + **	** + **	** + + + **	** + + **	** + **	** + + **	** + + **	** + + **
Alkaloid	** + **	** + **	** + + + **	** + + **	** + + + + + **	** + + **	** + + **	** + + **	** + **	** + + **
Tannins	** + **	** + **	** + + + **	** + **	** + + + **	** + + **	** + **	** + + + + **	** + **	** + **

−: absent (+, + +, + + + and + + + +) = indicate the Relative intensity of producing color.

**Table 3:** Total phenolic, flavonoids and alkaloid compounds (g/100 g) of different wild plants extracts.

Wild plants	Bioactive compounds					
	Phenolic compound		Flavonoid compounds		Alkaloid compounds	
	In extract	In plant	In extract	In plant	In extract	In plant
1. *C. monacantha*	1.78^i^ ± 0.006	0.80^ g^ ± 0.006	1.42^ g^ ± 0.015	0.61^b^ ± 0.021	2.41^a^ ± 0.110	0.32^hi^ ± 0.082
2. *S. plumosa*	0.80^j^ ± 0.000	0.30^j^ ± 0.000	0.60^j^ ± 0.006	0.21^ h^ ± 0.010	2.60^a^ ± 0.238	0.31^hij^ ± 0.144
3. *A. monosperma*	4.01^gh^ ± 0.015	0.43^ h^ ± 0.012	3.88^e^ ± 0.006	0.41^ef^ ± 0.012	2.21^a^ ± 0.038	2.59^c^ ± 0.030
4. Z. *arabicum*	6.68f. ± 0.006	1.19f. ± 0.006	1.35^gh^ ± 0.015	0.20^hi^ ± 0.000	2.60^a^ ± 0.037	0.44^ h^ ± 0.004
5. *T. hirsuta*	16.27^e^ ± 0.252	1.61^de^ ± 0.017	3.27f. ± 0.058	0.32^ g^ ± 0.015	2.58^a^ ± 0.094	1.02^efg^ ± 0.055
6. *B. indica*	23.06^b^ ± 0.051	3.03^b^ ± 0.058	4.75^c^ ± 0.042	0.55^c^ ± 0.010	2.47^a^ ± 0.167	1.39^e^ ± 0.156
7. *E. bonariensis*	19.45^c^ ± 0.046	8.41^a^ ± 0.006	4.87^b^ ± 0.006	2.14^a^ ± 0.006	2.48^a^ ± 0.005	2.39^ cd^ ± 0.043
8.* N. glauca*	18.00^d^ ± 0.000	1.64^d^ ± 0.000	4.50^d^ ± 0.000	0.41^e^ ± 0.000	2.71^a^ ± 0.163	7.01^a^ ± 0.163
9.* C. acutum*	117.19^a^ ± 0.190	1.80^c^ ± 0.006	29.10^a^ ± 0.173	0.50^d^ ± 0.006	2.24^a^ ± 0.773	3.59^b^ ± 0.797
10.* Z. album*	4.13^ g^ ± 0.058	0.40^hi^ ± 0.000	1.05^i^ ± 0.006	0.10^j^ ± 0.006	2.22^a^ ± 0.166	1.25^ef^ ± 0.156

Values are mean ± SD (n = 3). Means within the same column followed by different letters are significantly different (p < 0.05) according to Duncan's Multiple Range Test (DMRT). For the alkaloid (extract) column, the omnibus ANOVA was not statistically significant (p = 0.317); accordingly, no differentiating letters are assigned and all species share the same letter.

### Qualitative phytochemical profiling

Qualitative phytochemical screening of the ethanolic extracts from the collected wild plants revealed marked variation in the presence and relative abundance of secondary metabolites (Table 2). Phenolic compounds were detected in most of the examined extracts, with very strong intensity observed in *N. glauca* (plant no. 8, + + + +), followed by high levels in plants 3, 6, and 7 (+ + +), whereas phenolics were absent in plants 2, 4, and 10. Flavonoids were also widely distributed, showing very strong reactions in plants 3 and 8 (+ + + +), moderate to high intensities in plants 5, 7, and 9 (+ + to + + +), and weak or absent responses in a few samples. Proteins were detected in all extracts, generally at low to moderate levels, with plant 8 exhibiting the highest intensity (+ +).

Coumarins were present across all samples, with the strongest response recorded in plant 8 (+ + + +), while glycosides showed moderate to high abundance in most plants, particularly in plants 3 and 5 (+ + +). Alkaloids were detected in all extracts, with plant 5 showing a very strong reaction (+ + + + +), followed by high levels in plant 3 (+ + +). Tannins were also commonly observed, with pronounced intensities in plants 3, 5, and 8 (+ + + to + + + +). Overall, *N. glauca* demonstrated the richest phytochemical profile, exhibiting strong to very strong reactions for most of the screened constituents.

### Quantitative analysis of phenolics, flavonoids, and alkaloids

The quantitative analysis of total phenolic, flavonoid, and alkaloid contents in the examined wild plant extracts revealed significant variations among species (Table 3). Total phenolic compounds in the extracts ranged from 0.80 to 117.19 g/100 g, with *C. acutum* exhibiting the highest value (117.19 g/100 g), followed by *B. indica *(23.06 g/100 g), *E. bonariensis* (19.45 g/100 g), *N. glauca* (18.00 g/100 g), and *T. hirsuta* (16.27 g/100 g). The alkaloid content in the extracts showed a relatively narrow variation, ranging from 2.21 to 2.71 g/100 g, with the highest level detected in *N. glauca* (2.71 g/100 g). In contrast, alkaloid content in the plant material varied more widely, with *N. glauca* again exhibiting the highest concentration (7.01 g/100 g), followed by *C. acutum* (3.59 g/100 g) and *A. monosperma* (2.59 g/100 g).

Substantial variations between plant material and extract concentrations can be attributed to plant species variability, the nature and intensity of environmental stresses affecting each species, and the differential solubility of bioactive constituents in the extraction solvent (aqueous ethanol); variations in extraction efficiency (Fig. 3) likely also contributed.

### Correlation between phytochemical content and antioxidant activity

Pearson correlation coefficients were calculated between the total phenolic, flavonoid, and alkaloid content of each extract (Table 3) and its corresponding antioxidant activity in the DPPH, ABTS, KMnO₄, and DCPIP assays at 1000 µg/mL (Table 5) across the ten wild plant species (n = 10; Table 4). None of the twelve correlations reached statistical significance (all p > 0.05; |r| ranging from 0.16 to 0.45), most plausibly reflecting the pronounced ceiling/saturation effect at 1000 µg/mL, at which most extracts already scavenged more than 95% of the DPPH radical, compressing the dynamic range available to detect a linear association with any single compound class.

**Table 4:** Pearson correlation coefficients (r) between total phenolic, flavonoid and alkaloid content and antioxidant activity across ten wild plant extracts (n = 10; all p > 0.05).

Compound class	DPPH (r)	ABTS (r)	KMnO₄ (r)	DCPIP (r)
Total phenolics	0.23	−0.45	−0.28	−0.32
Total flavonoids	0.24	−0.41	−0.25	−0.37
Total alkaloids	−0.38	0.31	0.43	0.16

### Antioxidant activity of wild plants' maceration extracts

The antioxidant activity of the investigated wild plant extracts was evaluated using DPPH, ABTS, KMnO₄, and DCPIP assays at different concentrations (125–1000 µg/mL) (Table 5). Overall, all extracts exhibited concentration-dependent antioxidant activity across the four assays. *A. monosperma*, *T. hirsuta*, *E. bonariensis*, and *N. glauca* demonstrated the strongest DPPH inhibition at 1000 µg/mL, with values approaching or exceeding 99%. *Z.arabicum* exhibited the highest ABTS scavenging activity (91.87% at 1000 µg/mL), followed by *A. monosperma* and *S. plumosa*. In the KMnO₄ assay, strong responses (> 80%) were recorded for *A. monosperma*, T. hirsuta, *E. bonariensis*, and *N. glauca*. *Z. arabicum*, *T. hirsuta*, and *Z. album* showed the most pronounced DCPIP reduction (> 75%).

**Table 5:** Antioxidant activity (as %) of different wild plants extract against DPPH, ABTS, KMnO_4_ and DCPIP assays at different concentrations.

Wild plants	Conc. (ppm)	Antioxidant assay			
		DPPH	ABTS	KMnO_4_	DCPIP
*1. C. monacantha*	1000	98.65^fghijk^ ± 0.15	63.68^opq^ ± 0.28	73.61^ mn^ ± 0.42	63.92^ h^ ± 0.43
	500	97.70^lmno^ ± 0.36	43.31^hh^ ± 0.51	69.83^t^ ± 0.50	61.89^lmn^ ± 0.14
	250	83.98^aa^ ± 0.54	35.14^ mm^ ± 0.31	60.72ff. ± 0.38	61.73^lmnopqrs^ ± 0.16
	125	68.42^hh^ ± 0.93	19.25^nn^ ± 0.23	57.30^iijj^ ± 0.36	54.56^jj^ ± 0.47
*2. S. plumosa*	1000	77.33^ccdd^ ± 1.00	85.49^ cd^ ± 0.37	82.03^de^ ± 0.62	65.85^e^ ± 0.34
	500	72.85ff. ± 0.17	85.08^cde^ ± 0.21	74.80^kl^ ± 0.35	61.88^lmnop^ ± 0.18
	250	53.94^nn^ ± 0.38	65.35^jklm^ ± 0.14	70.77^pqr^ ± 0.29	60.47^uvw^ ± 0.42
	125	61.81^llmm^ ± 1.16	63.88° ± 0.51	65.65^aa^ ± 0.56	60.00^vwxyz^ ± 0.29
*3. A. monosperma*	1000	99.80^abc^ ± 0.07	85.56^c^ ± 0.46	90.47^a^ ± 0.50	62.26^jkl^ ± 0.74
	500	99.76^abcd^ ± 0.01	85.08^cdef^ ± 0.21	82.31^d^ ± 0.63	61.54^lmnopqrst^ ± 0.39
	250	99.38^abcdefg^ ± 0.33	65.48^jk^ ± 0.23	66.53^xyz^ ± 0.51	59.17^aabbccdd^ ± 0.42
	125	95.96^rs^ ± 0.76	63.84^op^ ± 0.46	40.29^nn^ ± 0.37	53.69^ll^ ± 0.25
*4. Z. arabicum*	1000	82.81^bb^ ± 0.70	91.88^a^ ± 0.13	70.76^pqrs^ ± 1.31	61.91^ lm^ ± 0.14
	500	70.84^gg^ ± 0.40	91.63^ab^ ± 0.45	68.68^uv^ ± 0.41	61.75^lmnopqr^ ± 0.19
	250	63.08^jjkk^ ± 0.76	75.39^ g^ ± 0.28	60.70^ffgg^ ± 0.37	59.03^bbccddee^ ± 0.35
	125	62.40^kkll^ ± 0.63	57.83^wx^ ± 0.62	57.45^ii^ ± 0.52	54.50^jjkk^ ± 0.47
*5. T. hirsuta*	1000	99.92^a^ ± 0.10	67.29^i^ ± 0.30	85.16^b^ ± 0.63	75.31^ab^ ± 0.49
	500	98.68^efghij^ ± 0.46	65.07^jklmn^ ± 0.20	74.84^ k^ ± 0.28	61.88^lmno^ ± 0.18
	250	97.62^lmnopq^ ± 0.48	60.39^t^ ± 0.17	70.81^pq^ ± 0.44	60.59^uv^ ± 0.60
	125	93.03^x^ ± 0.66	58.72^v^ ± 0.20	65.47^aabb^ ± 0.47	59.87^aavwxyz^ ± 0.15
*6. B. indica*	1000	98.73^efghi^ ± 0.25	65.43^jkl^ ± 0.41	75.89^ij^ ± 0.22	62.73^j^ ± 0.25
	500	94.35^vw^ ± 0.20	63.26^opqr^ ± 0.33	74.36^klm^ ± 0.24	62.72^jk^ ± 0.34
	250	94.49^uv^ ± 0.63	52.70^bbccdd^ ± 0.26	65.10^aabbcc^ ± 0.57	60.26^vwx^ ± 0.55
	125	91.04^y^ ± 0.58	45.76^gg^ ± 0.25	54.09^kk^ ± 0.19	59.34^aabbccz^ ± 0.61
*7. E. bonariensis*	1000	99.61^abcde^ ± 0.32	70.72^ h^ ± 0.28	83.30^c^ ± 0.21	64.81^ g^ ± 0.27
	500	97.98^ijklm^ ± 0.23	63.09^opqrs^ ± 0.41	79.51^ h^ ± 0.28	65.63^ef^ ± 0.55
	250	95.85st ± 0.18	59.58^u^ ± 0.34	72.54° ± 0.36	56.70^hhii^ ± 0.66
	125	95.24^stu^ ± 0.51	43.18^hhii^ ± 0.41	59.71^hh^ ± 0.34	51.40^nn^ ± 0.45
*8. N. glauca*	1000	99.90^ab^ ± 0.10	65.87^j^ ± 0.15	81.59^def^ ± 0.47	61.04^qrstu^ ± 0.45
	500	99.35^abcdefgh^ ± 0.30	58.15^vw^ ± 0.43	81.49^defg^ ± 0.41	58.61^ccddeeff^ ± 0.46
	250	99.46^abcdef^ ± 0.04	53.27^aabb^ ± 0.70	76.63^i^ ± 0.26	58.08^ffgg^ ± 0.10
	125	97.66^lmnop^ ± 0.48	50.79^ee^ ± 0.26	71.13^p^ ± 0.62	57.25^hh^ ± 0.43
*9. C. acutum*	1000	97.96^ijklmn^ ± 0.32	57.66^wxy^ ± 0.35	64.93^aabbccdd^ ± 0.24	61.75^lmnopq^ ± 0.34
	500	96.78^r^ ± 0.30	53.89^aa^ ± 0.09	69.10^tu^ ± 0.92	60.22^vwxy^ ± 0.77
	250	90.47^yz^ ± 0.40	53.05^bbcc^ ± 0.82	67.86^w^ ± 0.14	59.44^aabbz^ ± 0.43
	125	77.48^ cc^ ± 0.79	48.17ff. ± 0.39	63.22^ee^ ± 0.37	52.45^ mm^ ± 0.67
*10. Z. album*	1000	98.00^ijkl^ ± 0.63	57.20^xyz^ ± 0.43	66.94^x^ ± 0.37	75.47^a^ ± 0.42
	500	76.95^ccddee^ ± 0.07	40.64^kk^ ± 0.27	66.57^xy^ ± 0.23	73.47^c^ ± 0.50
	250	67.85^hhii^ ± 0.25	42.44^iijj^ ± 1.70	52.43^ mm^ ± 0.36	67.13^d^ ± 0.33
	125	63.64^jj^ ± 0.51	39.50^ll^ ± 0.50	53.49^kkll^ ± 0.39	63.86^hi^ ± 0.18
Ascorbic acid (natural Antioxidant standard)	1000	97.70 ± 0.21	100.00 ± 0.09	100.00 ± 0.21	97.94 ± 0.09
	500	96.01 ± 0.02	99.30 ± 0.21	99.93 ± 0.15	95.35 ± 0.10
	250	94.69 ± 0.21	99.20 ± 0.21	98.80 ± 0.35	95.30 ± 0.21
	125	94.15 ± 0.17	98.20 ± 0.14	98.80 ± 0.45	95.00 ± 0.33

Data are given as mean ± SE (n = 3). ^a, b and c^ Means within the same column with different letters are significantly different (p < 0.05).

A strong positive correlation was observed among the four antioxidant assays: ABTS vs. KMnO₄ (r = 0.92), ABTS vs. DCPIP (r = 0.93), and DPPH vs. KMnO₄ (r = 0.90).

### TLC bio-autography for antioxidant activity

Separation of wild plant extracts by TLC bioautography revealed the presence of antioxidant compounds in all tested extracts. Yellowish and white bands appearing against the purple background were indicative of strong antioxidant activity (Fig. 4). *A. monosperma*, *T. hirsuta*, and *N. glauca* exhibited particularly high levels of antioxidant compounds, with *N. glauca *displaying the most abundant antioxidant constituents among the studied plants.

### Antioxidant activity of semi-purified fractions of *N. glauca*

The semi-purified fractions of *N. glauca* exhibited considerable variation in antioxidant activity against DPPH and ABTS radicals, depending on fraction number and concentration (100–200 ppm) (Table 6). Fractions 9, 10, 11, and 8 showed the strongest DPPH activity, exceeding 94% at 200 ppm; fraction 16 showed negligible activity (< 5%). Fraction 11 achieved the highest ABTS inhibition (87.17% at 200 ppm), followed by fractions 10 (82.39%) and 9 (81.89%). Overall, fractions 8–11 were identified as the most potent antioxidant fractions, while fractions 14 and 16 exhibited the lowest activity in both assays.

**Table 6:** Antioxidant activity (as %) of semi-purified fractions of *N. glauca* against DPPH and ABTS assays at 100 and 200 ug/ml.

Fraction no	Conc. (ppm)	Against DPPH	Against ABTS	Fraction no	Conc. (ppm)	Against DPPH	Against ABTS
1	100	45.25^bb^ ± 0.17	49.68^t^ ± 0.26	9	100	98.10^efg^ ± 0.16	74.72^def^ ± 0.21
	200	99.90^a^ ± 0.21	47.80^v^ ± 0.36		200	98.16^ef^ ± 0.21	81.89^c^ ± 0.28
2	100	56.03^z^ ± 0.24	56.22^q^ ± 0.16	10	100	98.01^fgh^ ± 0.50	74.84^d^ ± 0.31
	200	56.50^y^ ± 0.19	57.23° ± 0.40		200	98.01^fghi^ ± 0.24	82.39^b^ ± 0.62
3	100	49.08^aa^ ± 0.14	40.25^y^ ± 0.23	11	100	94.33^ l^ ± 0.24	74.84^de^ ± 0.24
	200	73.48^u^ ± 0.09	42.76^w^ ± 0.09		200	98.58^bcd^ ± 0.24	87.17^a^ ± 0.21
4	100	5.33^ cc^ ± 0.00	49.68^tu^ ± 0.05	12	100	90.92^q^ ± 0.17	57.74^n^ ± 0.59
	200	5.20^ccdd^ ± 0.00	54.84^r^ ± 0.24		200	94.13^lmno^ ± 0.24	58.49^ m^ ± 0.14
5	100	60.50^w^ ± 0.29	42.14^x^ ± 0.16	13	100	94.33^ lm^ ± 0.36	29.25^aa^ ± 0.19
	200	67.52^v^ ± 0.21	57.23^op^ ± 0.21		200	98.87^b^ ± 0.14	38.44^z^ ± 0.24
6	100	74.47^t^ ± 0.19	67.30^ h^ ± 0.38	14	100	94.26^lmn^ ± 0.19	12.15ff. ± 0.28
	200	84.11^ s^ ± 0.23	68.55^ g^ ± 0.00		200	98.82^bc^ ± 0.12	24.88^ cc^ ± 0.16
7	100	90.07^r^ ± 0.14	66.92^hijk^ ± 0.33	15	100	58.44^x^ ± 0.23	27.95^bb^ ± 0.09
	200	94.04^lmnop^ ± 0.23	67.30^hi^ ± 0.21		200	96.88^j^ ± 0.17	53.54^ s^ ± 0.31
8	100	94.89^ k^ ± 0.28	64.78^ l^ ± 0.35	16	100	5.10^ccddee^ ± 0.00	13.92^ee^ ± 0.07
	200	98.44^de^ ± 0.16	67.30^hij^ ± 0.23		200	4.90^ddeeff^ ± 0.00	20.87^dd^ ± 0.16

Values are mean ± SD (n = 3). Means within the same assay column (DPPH or ABTS), compared across all 16 fractions and both concentrations, followed by different letters are significantly different (p < 0.05) according to Duncan's Multiple Range Test (DMRT).

### GC–MS/MS characterization of fraction 11

**Fig. 5:**
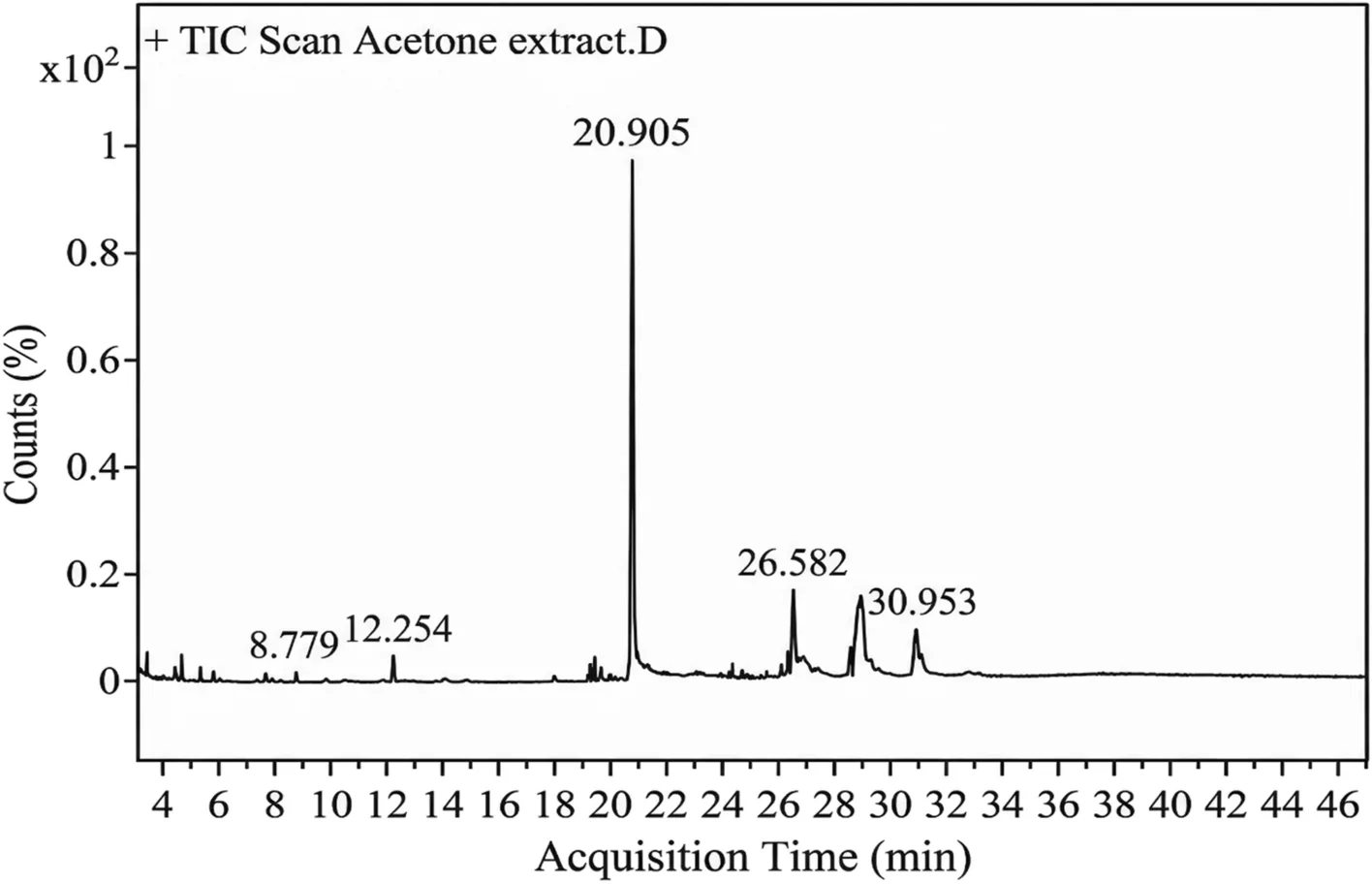
GC/MS/MS chromatogram of fraction no. 11 of *N. glauca*.

The chemical composition of Fraction 11 was investigated using gas chromatography–tandem mass spectrometry (GC/MS/MS); the analysis is presented in Fig. 5 and Table 7. The GC/MS/MS data revealed a complex chemical profile comprising six distinct phytocomponents. Among these, the major constituents were identified as Anabasine (39.26%), Nornicotine (36.50%), cyclohexanone,2-((4-methoxyphenyl) piperidinyl methyl) (10.02%), and Edetol (9.80%).

**Table 7:** Active ingredients as relative percentage of fraction no. 11 of *N. glauca* analyzed using GC/MS/MS.

SN	Rt (min)	Chemical compounds	Chemical structure	Mass	Retention index	Relative percentage
1	**4.66**	Xylene		**28.0**	**215**	**2.90**
				**91.1**		
2	**12.25**	Morpholine,4-(1-hydroxypropyl)		**28.0**	**847**	**1.55**
				**70.1**		
				**100.1**		
3	**20.90**	Anabasine		**28.1**	**760**	**39.26**
				**84.1**		
				**133.1**		
4	**26.58**	Edetol		**28.1**	**972**	**9.80**
				**70.1**		
				**102.1**		
				**146.1**		
				**202.1**		
5	**28.83**	Nornicotine		**28.1**	**1151**	**36.50**
				**59.1**		
				**98.1**		
				**146.1**		
				**204.2**		
				**260.2**		
				**361.3**		
6	**30.953**	Cyclohexanone,2((4-methoxyphenyl)) piperidinylmethyl))		**28.1**	**900**	**10.02**
				**59.1**		
				**130.1**		
				**204.2**		
				**262.2**		
				**305.3**		
				**361.3**		
				**419.3**		

### Antioxidant activity of sub-fractions obtained from fraction no. 11 of *N. glauca*

**Fig. 6:**
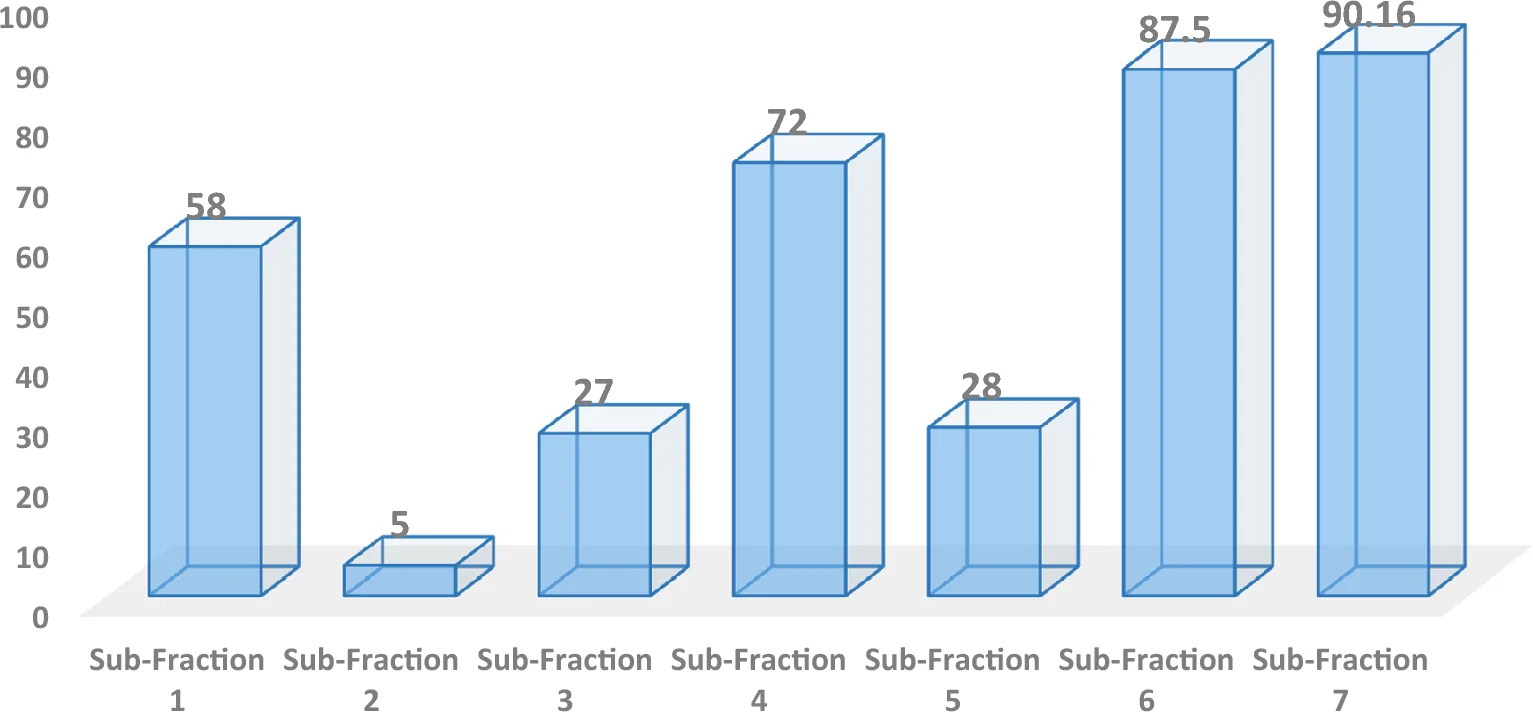
Antioxidant activity of sub-fractions collected from the column fractionation of fraction no. 11 from *N. glauca*.

The antioxidant activity of the sub-fractions obtained from the column fractionation of Fraction 11 was evaluated (Fig. 6). Sub-fraction 7 exhibited the highest antioxidant activity at 90.16%, followed closely by sub-fraction 6 with 87.5% inhibition. Sub-fraction 4 also demonstrated significant activity at 72%, whereas sub-fractions 1 and 5 showed moderate activity at 58 and 28%, respectively. Sub-fractions 3 and 2 displayed the lowest activity, with 27 and 5%, respectively.

### Combination index (CI) for antioxidant activity

The antioxidant interactions between the crude *N. glauca* extract and two isolated pure compounds (A and B) were evaluated using the Combination Index (CI) method (Table 8). Compound A (N,N-methylene-bis(14-docosene)) and Compound B (14-methylhexadecanoic acid) were identified as the primary constituents of subfractions 1 and 7, respectively. Consistent with established classification criteria^43^, a CI value below 0.8 indicates synergism, a value between 0.8 and 1.2 suggests an additive effect, and a value exceeding 1.2 signifies antagonism. The combination of the crude extract with Compound A exhibited a pronounced synergistic effect (CI = 0.53), indicating that the antioxidant activity of the mixture surpasses the sum of the activities of the individual components. In contrast, the combination of the crude extract with Compound B showed a strong antagonistic effect (CI = 28.8), suggesting that Compound B substantially inhibits the antioxidant activity of the crude extract, potentially through competitive inhibition of active antioxidant components or induction of pro-oxidant effects under the tested conditions.

**Table 8:** Combination index (CI) Values as Antioxidant and anticancer as IC_50_ (μg/mL)] of crude extract of *N. glauca* and its fractionated products on different Cancer cell lines.

Assay type	Cells	Sample	IC_50_ (μg/mL) ± SD	CI value	Nature of interaction
Antioxidant	N/A	*N. glauca* crude extract	63.99^b^ ± 0.476	0.53	Synergistic
		Pure compound A N, N-Methylene-Bis (14-Docosene)	120.517^a^ ± 10		
Antioxidant	N/A	*N. glauca* crude extract	63.99^b^ ± 0.476	28.8	Antagonistic
		Pure Compound B 14-Methylhexadecanoic acid	2.218^c^ ± 0.68		
Anticancer	_Hela_	*N. glauca* crude extract	146.04^b^ ± 0.5	0.139	Synergistic
		Pure compound A N, N-Methylene-Bis (14-Docosene)	1048.31^a^ ± 21.33		
Anticancer	_Hela_	*N. glauca* crude extract	146.04^b^ ± 0.5	2.2	Antagonistic
		Pure Compound B 14-Methylhexadecanoic acid	66.19^c^ ± 0.68		
Anticancer	_HepG2_	*N. glauca* crude extract	169.38^b^ ± 1.27	0.158	Synergistic
		Pure compound A N, N-Methylene-Bis (14-Docosene)	1071.54^a^ ± 23.15		

CI calculations are based on the individual IC50 values of the crude extract and the pure compound. The nature of sample interaction was identified as synergistic if CI < 0.8; as antagonistic if CI > 1.2; and as additive if CI ranges from 0.8 to 1.2.

These findings underscore the importance of understanding interactions among components in complex natural extracts. The synergistic effect observed with Compound A highlights the potential for developing enhanced antioxidant formulations by strategically combining specific isolated compounds with the crude extract, while the strong antagonism associated with Compound B emphasizes the need for careful evaluation of potential negative interactions when isolating and combining natural compounds for antioxidant applications.

### Suggested chemical structure of the bioactive constituent in the subfraction no. 1 and 7 of *N. glauca* (compounds* A and B)*

Subfractions 1 and 7 were selected for purification of the active constituents due to the high yield of subfraction 1 and the strongest antioxidant activity observed in subfraction 7. Preparative thin-layer chromatography (TLC) was employed to isolate the two compounds from these subfractions.

**Fig. 7:**
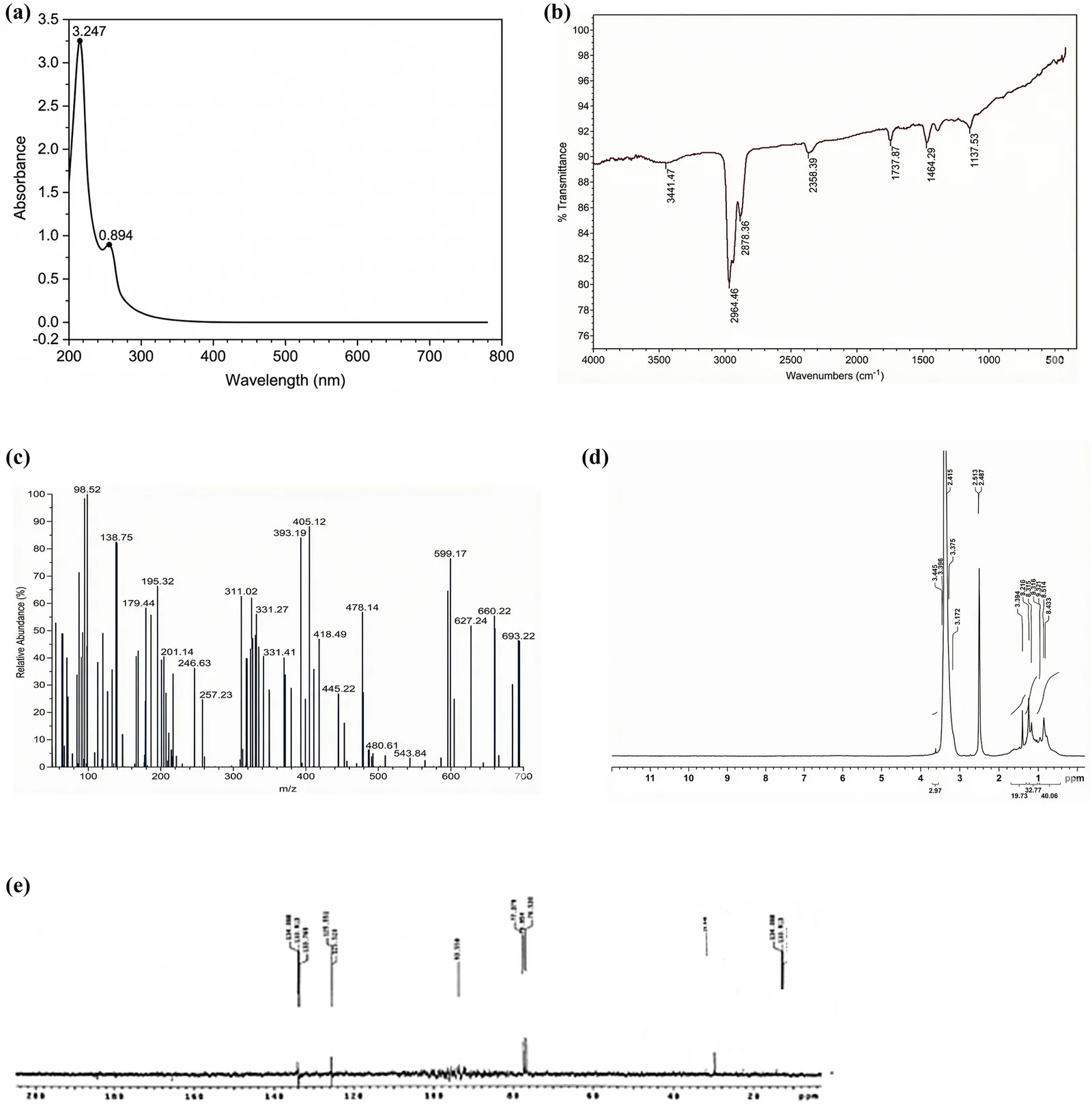
(**a**) UV scanning of fraction no. 1 (sub column from fraction no. 11) of *N. glauca*, (**b**) FTIR of fraction no. 1(sub column from fraction no. 11) of *N. glauca*, (**c**) Mass spectrum of fraction no. 1 (sub-column from fraction no. 11) of *N. glauca*, (**d**) 1H-NMR of fraction no. 1 (sub-column from fraction no. 11) of *N. glauca*, (**e**) 13C-NMR of fraction no. 1 (sub-column from fraction no. 11) of *N. glauca*.

Figure 7a–e illustrate the proposed chemical structure of the active constituent from subfraction 1, supported by comprehensive spectroscopic analyses (UV, IR, mass spectrometry, ^1^H-NMR, and ^13^C-NMR). The isolated compound was obtained as an amorphous white powder (yield 140 mg), freely soluble in hexane. The UV spectrum showed a maximum absorption (λ_max) at 230 nm (Fig. 7a). The IR spectrum (Fig. 7b) revealed a strong N–H stretching at 3420 cm⁻^1^, indicating a primary amine group; peaks at 2964 cm⁻^1^ and 2876 cm⁻^1^ correspond to C–H stretching of alkyl groups (–CH₂ and –CH₃), while the absorbance at 1737 cm⁻^1^ indicates the presence of a double bond, and a peak at 1130 cm⁻^1^ suggested a halogen group. Mass spectrometry (Fig. 7c) showed a molecular ion at m/z = 662, consistent with the molecular formula C₄₅H₉₄N₂. In the ^1^H-NMR spectrum (CDCl₃, 270 MHz) (Fig. 7d), methyl singlets were observed at δ 0.70 and 1.01 ppm, protons associated with the double bond or amino group appeared at δ 2.50 ppm, and signals corresponding to protons bound to the halogen group were observed at δ 3.2 and 3.4 ppm. The ^13^C-NMR spectrum (Fig. 7e) confirmed five distinct carbon environments: methylene and methyl carbons at δ 29.54 ppm, the carbon bound to oxygen at δ 76.95 ppm, and the double bond carbon at δ 125.52 ppm. Structure confirmation was further supported by comparison with data from the NIH database (National Library of Medicine) and the Spectral Database for Organic Compounds (SDBS). Based on these analyses, the compound from subfraction 1 was identified as N, N-methylene-bis(14-docosene) (Fig. 9).

**Fig. 8:**
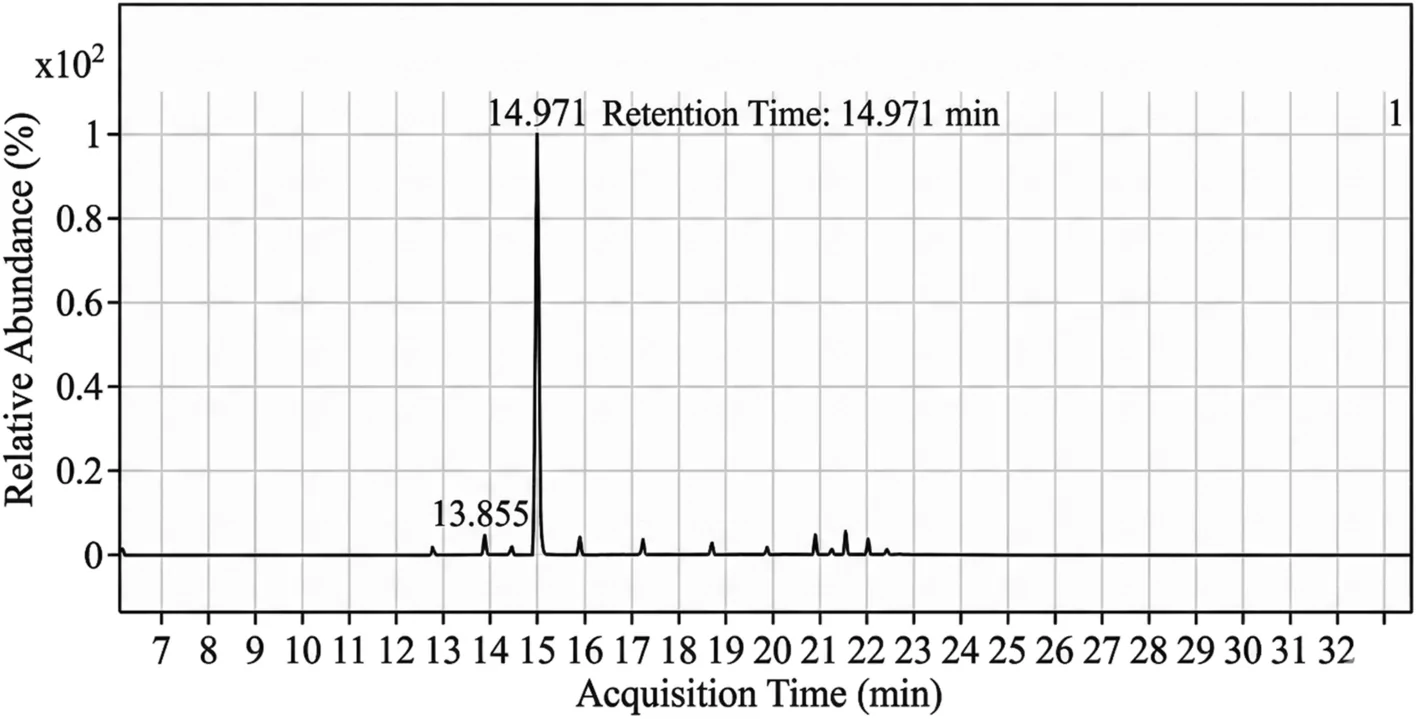
GC/MS/MS chromatogram of subfraction no. 7 of *N. glauca*.

**Fig. 9:**
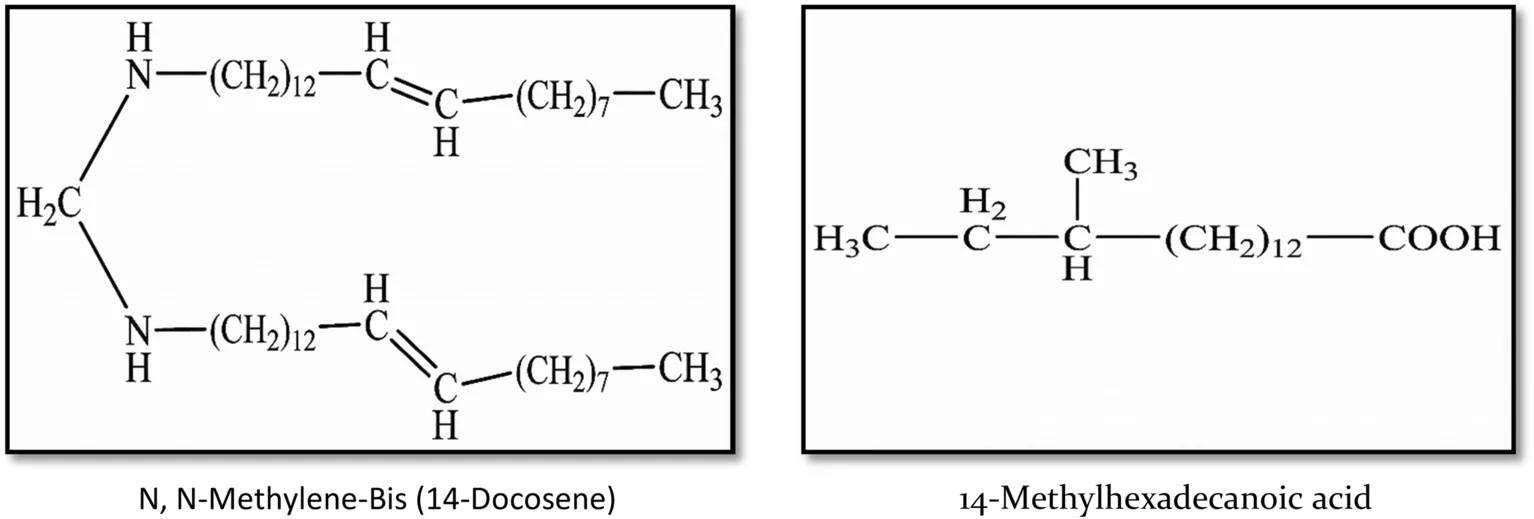
Suggested chemical structure of pure compounds separated from sub-fractions no. 1 and 7 of *N. glauca*.

The main compound isolated from subfraction 7 was characterized using GC–MS/MS after sub-column chromatography (Fig. 8) and identified as 14-methylhexadecanoic acid, molecular weight 270.5 g/mol, molecular formula C₁₇H₃₄O₂ (Fig. 9). This compound has been reported previously in *N. glauca* and other medicinal plants^44^, and its antioxidant potential has been confirmed in earlier studies^45–47^.

### Cytotoxicity assessment of crude extract and fractions

**Fig. 10:**
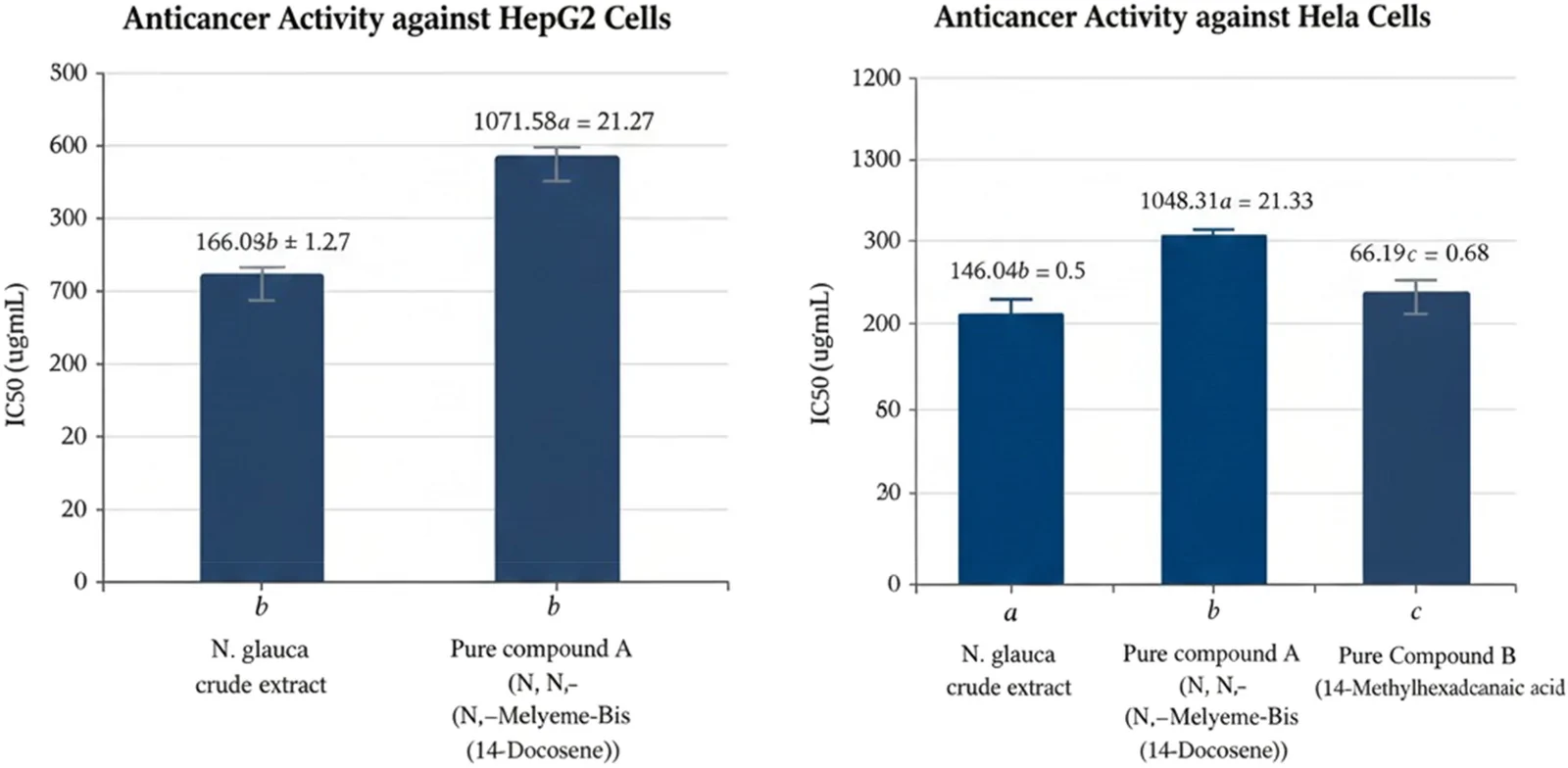
Anticancer as IC*50* (μg/mL) of crude extract of *N. glauca* and its pure compounds on different Cancer cell lines.

Figure 10 illustrates the anticancer activity of the crude *N. glauca* extract and its isolated compounds (A and B) against three cancer cell lines (HeLa, HepG2, and MCF7), expressed as IC₅₀ values (µg/mL). Compound B exhibited the highest potency against HeLa cells (IC₅₀ = 70 µg/mL), followed by the crude extract (150 µg/mL), whereas Compound A showed the lowest activity (IC₅₀ = 1000 µg/mL). A similar trend was observed in HepG2 cells. Both the crude extract and Compound A exhibited markedly reduced activity against MCF7 cells (IC₅₀ = 350,000 and 37,000 µg/mL, respectively), highlighting a cell-line-specific variation in sensitivity.

Under identical assay conditions, the reference anticancer drug doxorubicin exhibited markedly greater potency, with IC₅₀ values of 0.88 ± 0.02 µg/mL against HeLa cells and 0.91 ± 0.04 µg/mL against HepG2 cells, several orders of magnitude lower than those of the crude extract and isolated compounds. This confirms the sensitivity of the assay system and provides a validated pharmacological benchmark against which the natural-product IC₅₀ values reported here can be interpreted.

Fraction 11 displayed moderate cytotoxicity across all three cell lines, with IC₅₀ values of 876.98 µg/mL (HeLa), 486.76 µg/mL (HepG2), and 815.39 µg/mL (MCF7). The isolated Compound A showed the least cytotoxic activity across all tested cell lines (Table 9), indicating that this compound alone is unlikely to account for the cytotoxic effects observed in the crude extract and Fraction 11; the Combination Index analysis below suggests instead a possible synergistic contribution from Compound A when combined with other extract constituents — a point discussed further under Combination Index for Anticancer Activity below. Notably, high cell viability at lower concentrations was observed for the HepG2 cell line across all tested samples, indicating potential selective cytotoxicity.

**Table 9:** IC50 (μg/mL) of Compound B 14-Methylhexadecanoic acid on normal cell lines.

Normal cell lines	IC_50_ (μg/mL) ± SD	Cancer cell line	IC50 (μg/mL) ± SD
Wi38	328.77 ± 1.88	Hela	66.19^c^ ± 0.68
A549	146.23 ± 1.78		

### Combination index (CI) for anticancer activity

The enhanced potency of the crude *N. glauca* extract against HeLa and HepG2 cell lines compared to Fraction 11 and Compound A may be attributed to an auto-synergistic effect among the various components within the crude extract (Table 8). To investigate this, the Combination Index (CI) method was employed to evaluate the effects of combining the crude extract with the two isolated pure compounds (A and B) on HeLa and HepG2 cell lines.

In HeLa cells, the combination of the crude extract with Pure Compound A resulted in a synergistic interaction, with a CI value of 0.139, indicating that the combined effect of the crude extract and Compound A is greater than the sum of their individual effects. Conversely, the combination of the crude extract with Pure Compound B exhibited an antagonistic interaction (CI = 2.2), implying that these two components interfere with each other's anticancer activity in HeLa cells. A synergistic effect was similarly observed between the crude extract and Pure Compound A in HepG2 cells, with a CI value of 0.158, further supporting the potential of combining the crude extract with Compound A to achieve superior anticancer activity against hepatocellular carcinoma cells.

It is important to clarify that Compound A is not the most potent cytotoxic agent in isolation — its stand-alone IC₅₀ values in HeLa and HepG2 cells are consistently higher (i.e., weaker) than those of the crude extract (Fig. 10, Table 8). Rather, Compound A's significance lies in its capacity to potentiate the crude extract's activity when combined, as reflected in the strongly synergistic CI values obtained for both antioxidant (CI = 0.53) and anticancer (CI = 0.139 in HeLa; CI = 0.158 in HepG2) combinations. Compound A is therefore best understood throughout this study as a key synergy partner within the crude extract's phytochemical matrix, rather than as its principal active molecule in an absolute, stand-alone sense; the crude extract's superior activity likely reflects the cooperative contribution of multiple co-occurring constituents, consistent with the antioxidant fractionation data (Tables 6 and 8).

### Gene expression analysis

Gene expression analysis revealed that treatment of HeLa cells with Compound A significantly modulated the expression of key apoptotic regulatory proteins. Specifically, the pro-apoptotic gene Bax was markedly upregulated (5.133 ± 0.309-fold change relative to control), while the anti-apoptotic gene Bcl-2 was sharply downregulated (0.309 ± 0.01-fold change relative to control). In untreated control cells, the Bax/Bcl-2 ratio was approximately 1.0, indicating a balanced apoptotic threshold.

Compound A treatment was associated with a substantial shift in this balance, increasing the Bax/Bcl-2 ratio to approximately 16.6-fold relative to control — a finding consistent with the initiation of apoptosis, or programmed cell death.

An elevated Bax/Bcl-2 ratio is widely recognized as a key trigger of the mitochondrial pathway of apoptosis in various cancer cell types. The observed increase in Bax and decrease in Bcl-2 are consistent with a possible mitochondrial-centric mechanism of action for Compound A, potentially disrupting the mitochondrial outer membrane potential — a crucial early event in apoptosis — and plausibly promoting release of pro-apoptotic factors such as cytochrome c with subsequent activation of the caspase cascade. These mitochondrial and caspase-cascade steps were not directly measured in this study and are proposed based on the Bax/Bcl-2 data; direct confirmation (Annexin V/PI, caspase-3/7 activity, PARP cleavage, or TUNEL assays) is required before apoptosis induction can be considered established.

### Increased oxidative stress: malondialdehyde (MDA) levels

To further investigate the role of oxidative stress, MDA levels — a well-established biomarker of lipid peroxidation and oxidative damage — were measured. Compound A treatment resulted in a significant increase in MDA levels (14.868 ± 0.56-fold change relative to the control's FLD value, with a corresponding IC₅₀ of 2.539 µM).

This substantial elevation in MDA is consistent with, though not direct proof of, Compound A being associated with significant oxidative stress in HeLa cells, contributing to cellular damage and potentially playing a role in its anticancer efficacy. MDA is a downstream lipid-peroxidation biomarker and not a direct measure of intracellular reactive oxygen species (ROS); ROS-specific probes (e.g., DCFDA fluorescence or flow-cytometric ROS assays) would be needed to confirm oxidative stress directly. The potent cytotoxic effect of Compound A, as indicated by its low IC₅₀ value, further underscores its potential as an anticancer agent.

### Molecular docking of anabasine with target proteins (2Q79 and E6)

Because HeLa cells are HPV18-positive rather than HPV16-positive, the docking analysis below is presented as an exploratory assessment of anabasine's potential activity against high-risk HPV oncoproteins as a structural class, rather than as a direct mechanistic explanation for the cytotoxicity observed in the HeLa cytotoxicity assays reported above.

The molecular docking studies elucidated the interaction dynamics of anabasine with the active sites of two target proteins: 2Q79 (crystal structure of single chain E2C from HPV16) and E6 (7UAJ) (crystal structure of apo HPV16 E6), Furthermore, molecular docking against the HPV18 E6 domain (6SJV) was included in the evaluation to complement the HPV16 analysis, with all corresponding binding scores compiled (Tables 10 and 11).

**Table 10:** The docking results based on the binding affinity (score) of Anabasine docked into 2Q79, E6 (7UAJ), and the interaction between Anabasine and the active site in Hela (2Q79 and 7UAJ).

Target protein	Binding affinity/score (kcal/mol)
HPV16 E6 oncoprotein (7UAJ)	− 7.2349
HPV16 E2 protein (2Q79)	− 7.4638
HPV18 E6 domain (6SJV)	− 5.64

Values against 7UAJ and 2Q79 are reported as originally generated; please confirm these were obtained using the same docking.

**Table 11:** Predicted binding affinity scores (kcal/mol) of the studied phytochemicals against HPV16 and HPV18 targets.

Ligand name	HPV16 E6 Oncoprotein (7UAJ)	HPV16 E2 Protein (2Q79)	HPV18 E6 Domain (6SJV)	Rotatable Bonds (TORSDOF)
N, N-Methylene-Bis (1,14-Docosene)	−7.4	−3.3	− 5.59	Moderate
14-Methylhexadecanoic acid	−7.1	−4.0	− 4.88	44 (High)

(The binding affinity for 2Q79 reflects the interaction with the E2 DNA-binding domain).

Docking results revealed that anabasine exhibits a strong and stable interaction with the active site of 2Q79, as evidenced by a binding energy of − 7.4638 kcal/mol — consistent with established literature, where similar ligands (e.g., nicotine derivatives targeting related protein families) have demonstrated binding energies within the − 6 to − 8 kcal/mol range. The interaction is characterized by hydrogen bonds between anabasine and residues Lys349 and Ile350, which are pivotal for anchoring the ligand and contribute to the specificity and stability of the ligand–protein complex, complemented by hydrophobic interactions with Leu345. A comparable binding profile was obtained for the HPV16 E6 oncoprotein (7UAJ), with a binding energy of − 7.2349 kcal/mol.

Against the HPV18 E6 domain (6SJV), anabasine yielded a binding energy of − 5.64 kcal/mol — a moderately weaker interaction relative to HPV16 targets (2Q79: − 7.4638 kcal/mol; 7UAJ: − 7.2349 kcal/mol) (Table 10). All three docking runs were performed under identical AutoDock Vina parameters (exhaustiveness = 56), allowing the scores to be directly compared across targets.

**Fig. 11:**
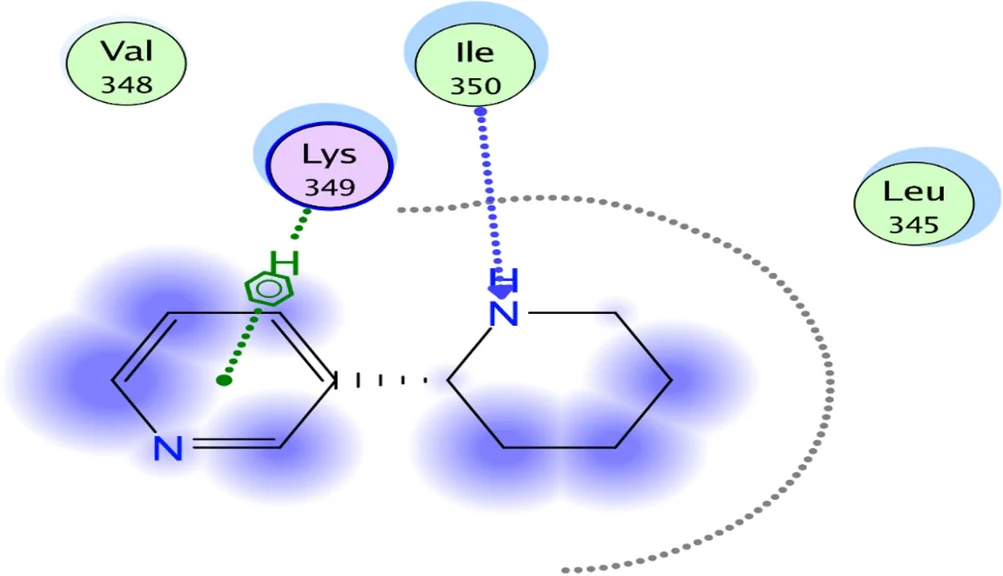
2D of Anabasine showing its interaction with the active site.

**Fig. 12:**
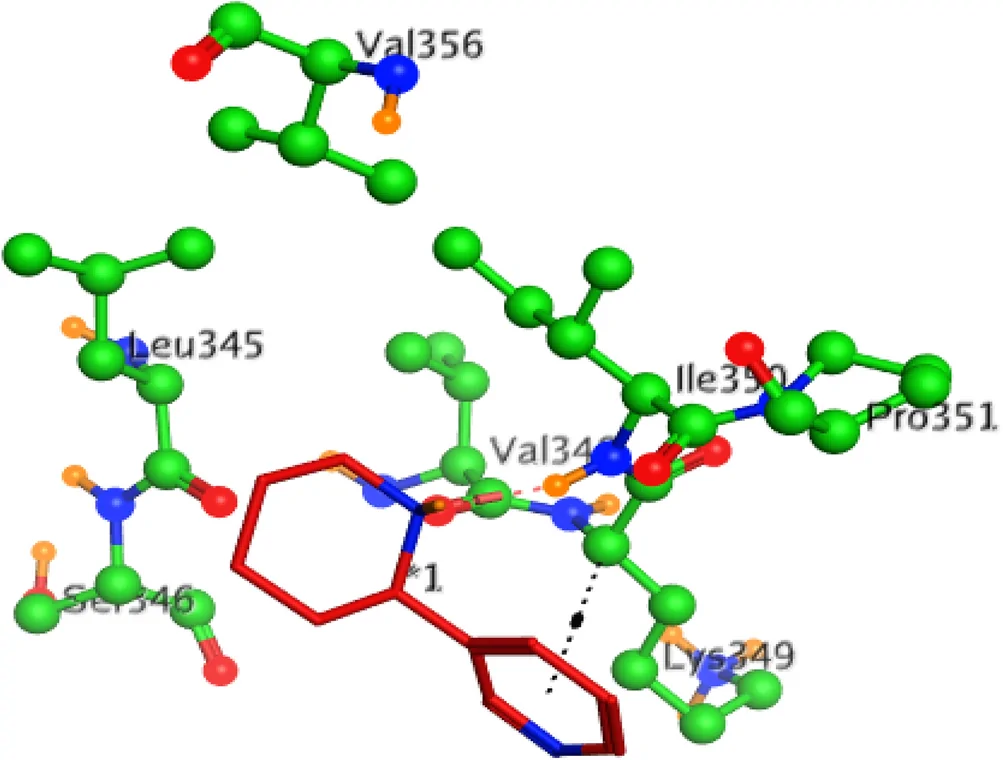
3D of Anabasine showing its interaction with the 2Q79 active site.

**Fig. 13:**
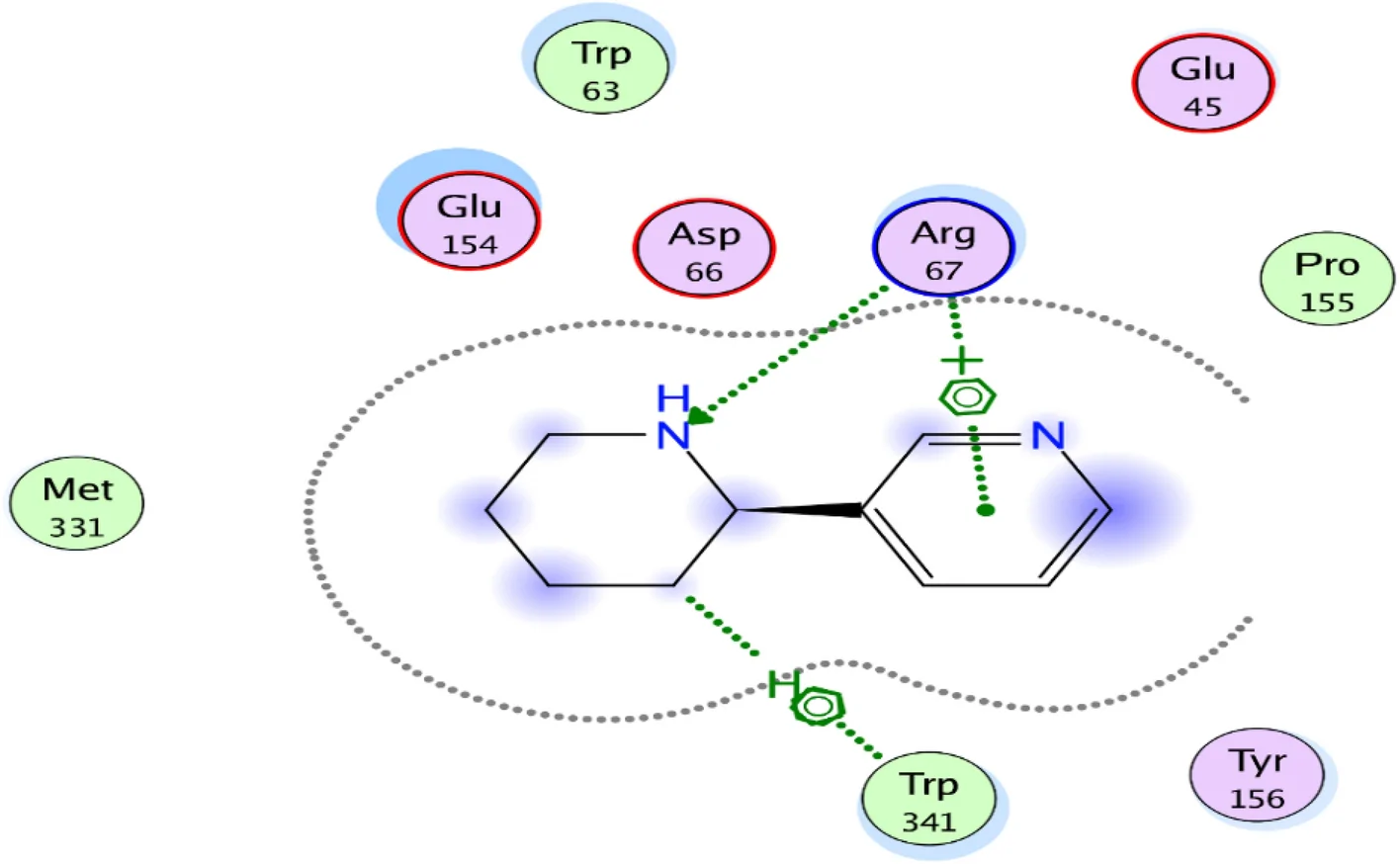
2D of Anabasine showing its interaction with the active site.

**Fig. 14:**
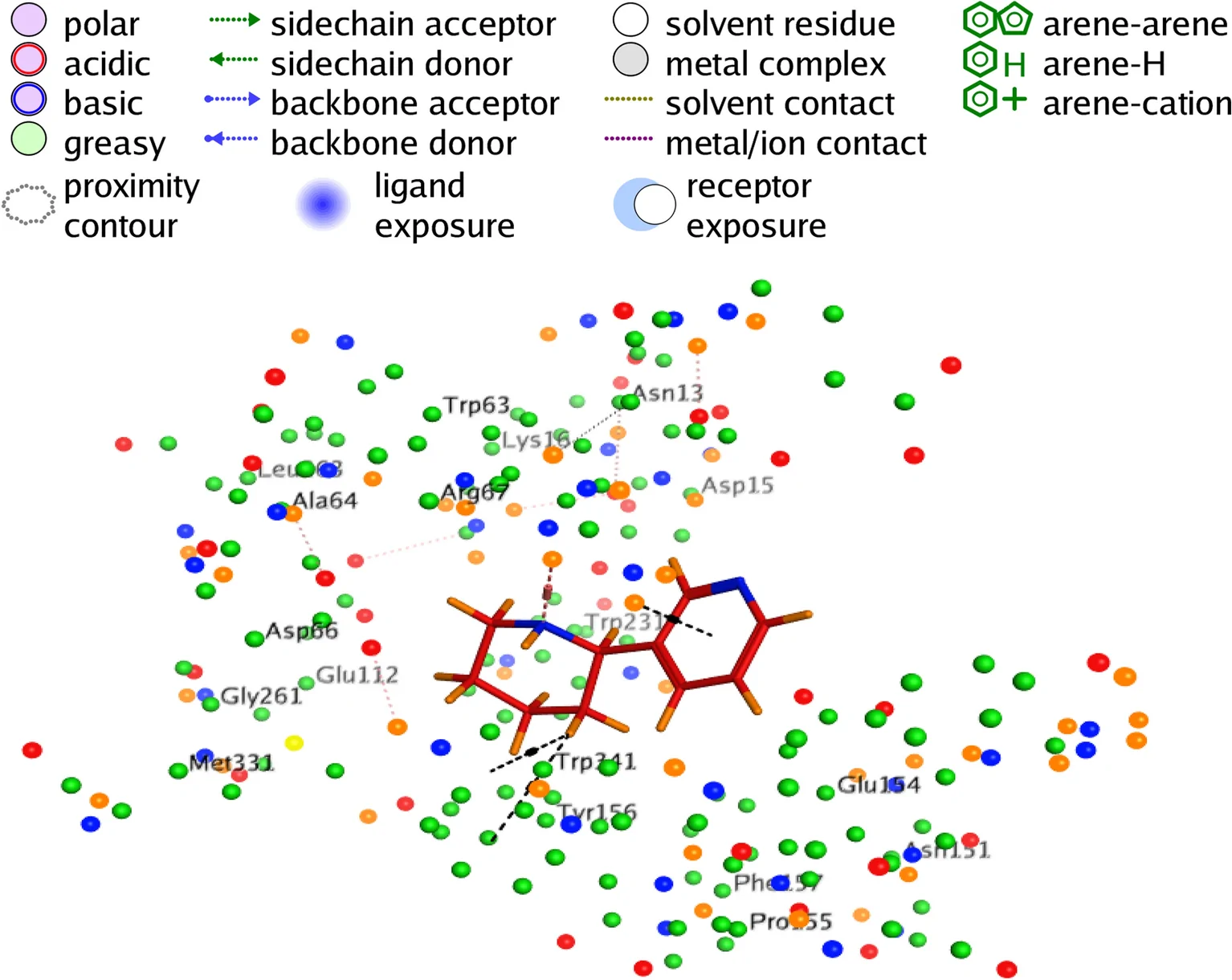
3D of Anabasine showing its interaction with the active site.

These findings provide computational support consistent with the promising therapeutic potential of *N. glauca* extract and its active compounds as antioxidant and anticancer agents, highlighting their potential molecular mechanisms of action. A combination of hydrogen bonding, hydrophobic interactions, and favourable spatial orientation mediates anabasine's affinity for both 2Q79 and E6 (7UAJ); van der Waals forces and π-π stacking interactions also contribute to the stability of the ligand–protein complexes. These detailed interaction profiles (Figs. 11–14) are computational predictions that support the potential of anabasine as a candidate for drug development targeting these proteins, pending experimental validation.

These docking results should be interpreted as computational predictions of binding affinity and pose, providing hypothesis-generating support for anabasine's potential interaction with HPV16 E2 and E6 oncoproteins; they do not constitute direct experimental evidence of ligand–protein binding, and complementary biophysical or cell-based validation is required before mechanistic conclusions can be drawn.

### Molecular Docking and structure–activity relationship (SAR) analysis

**Fig. 15:**
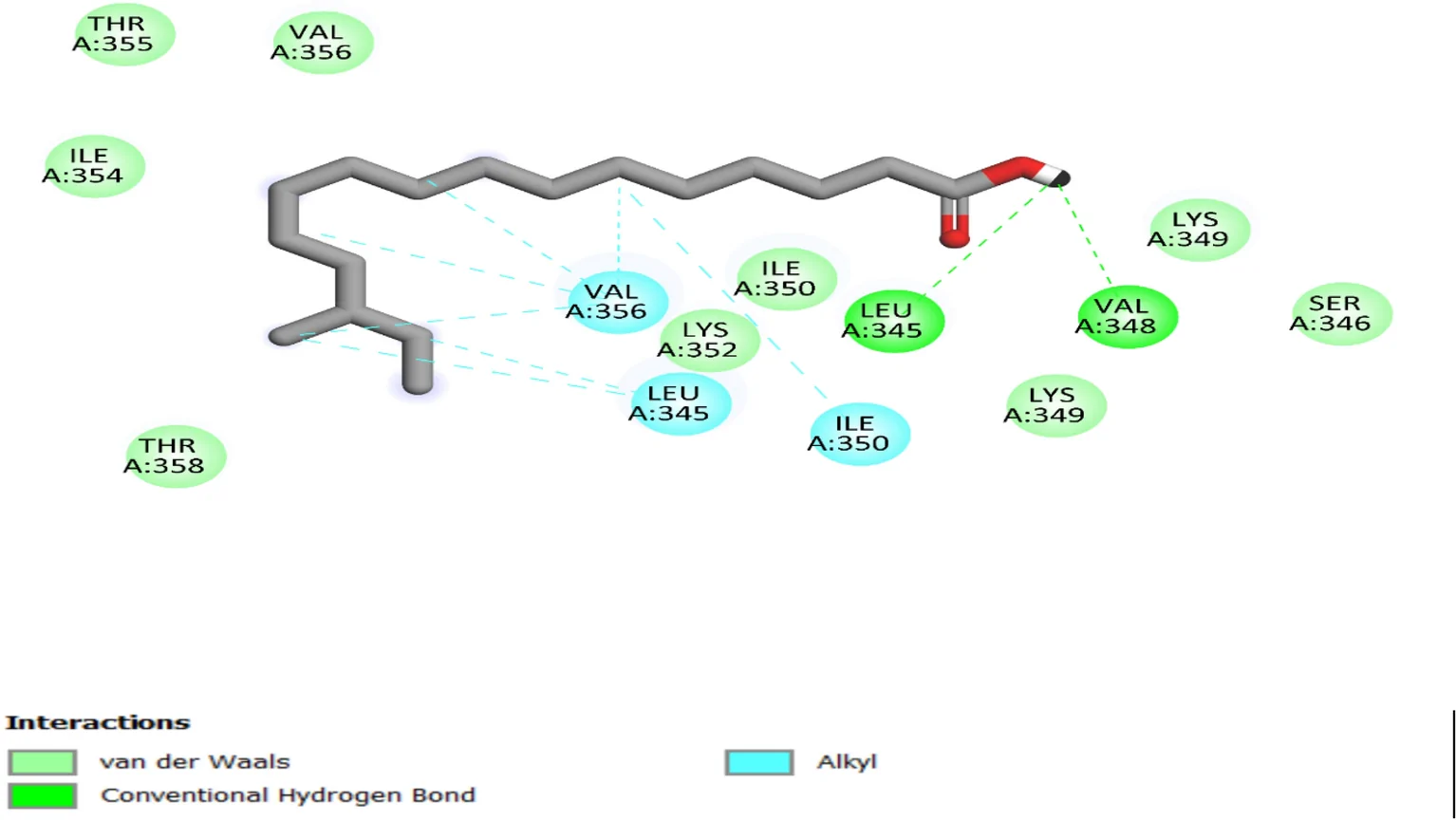
2D interaction profile of the 14-Methylhexadecanoic acid ligand docked into the binding pocket of HPV16 E2 active pocket (PDB: 2Q79).

**Fig. 16:**
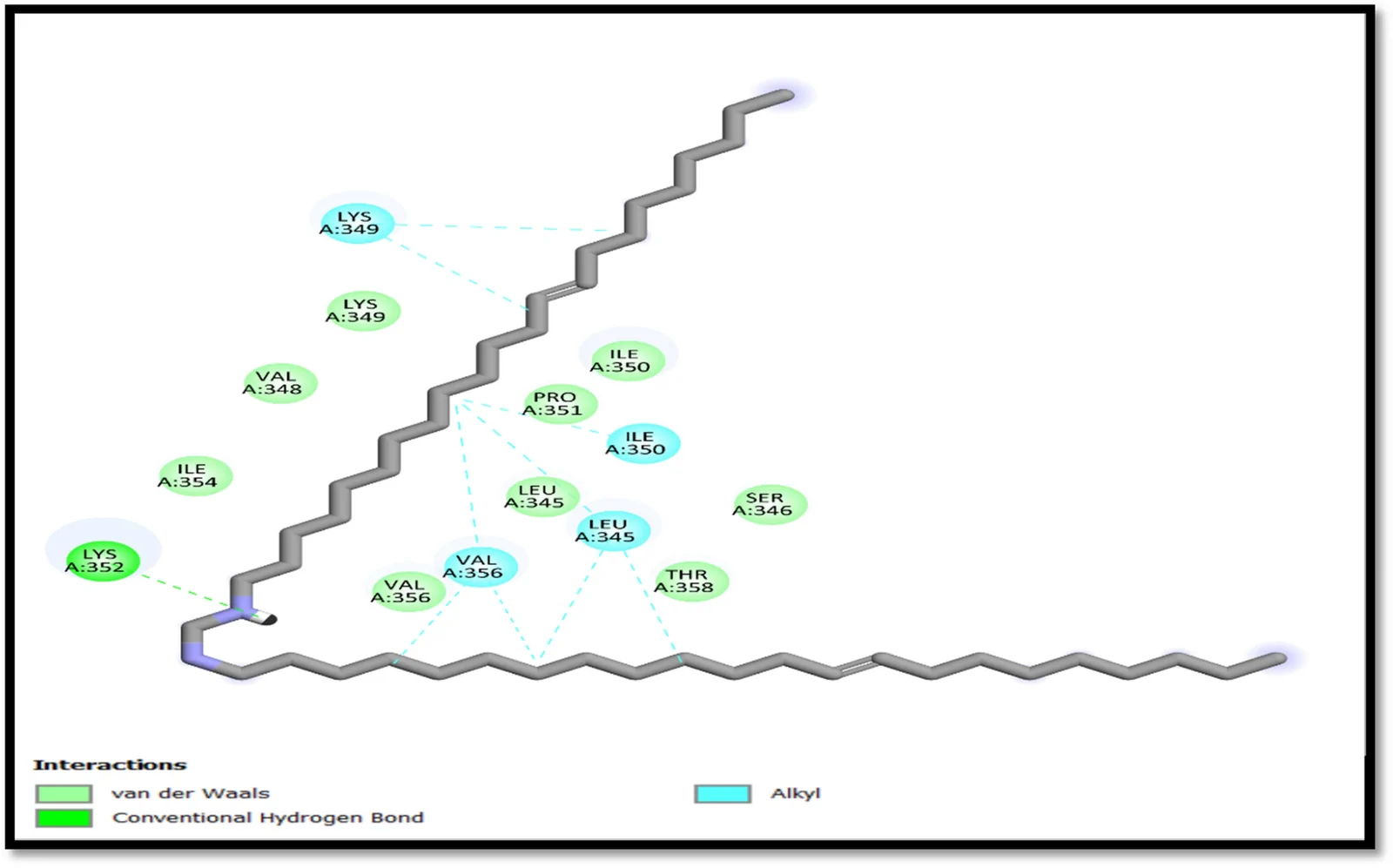
2D interaction map of the N, N-Methylene-Bis (14-Docosene) ligand docked within the binding site of (HPV16 E2 active pocket (PDB: 2Q79).

**Fig. 17:**
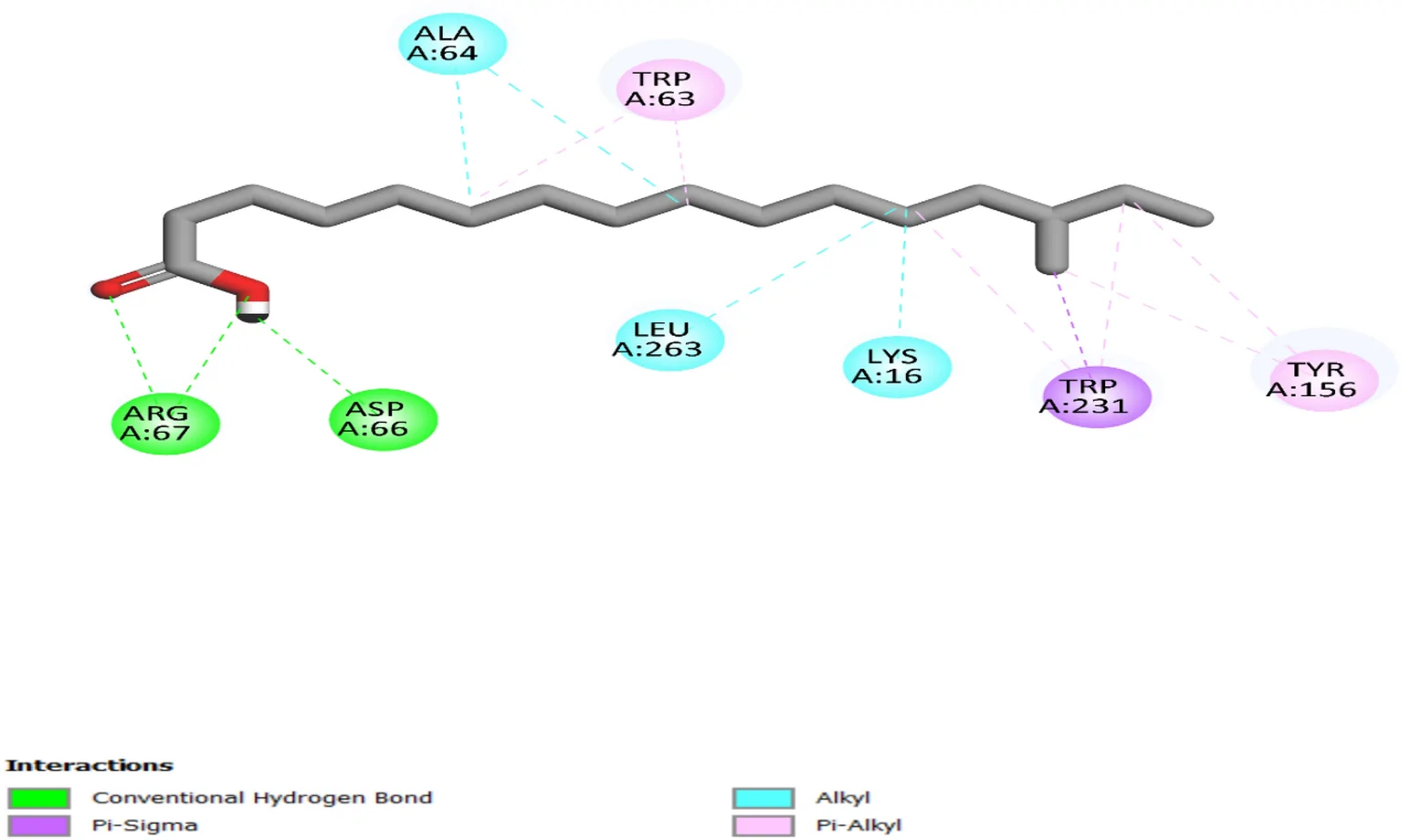
2D interaction diagram of the 14-Methylhexadecanoic acid ligand docked into the active site of the HPV16 E6 protein (PDB ID: 7UAJ).

**Fig. 18:**
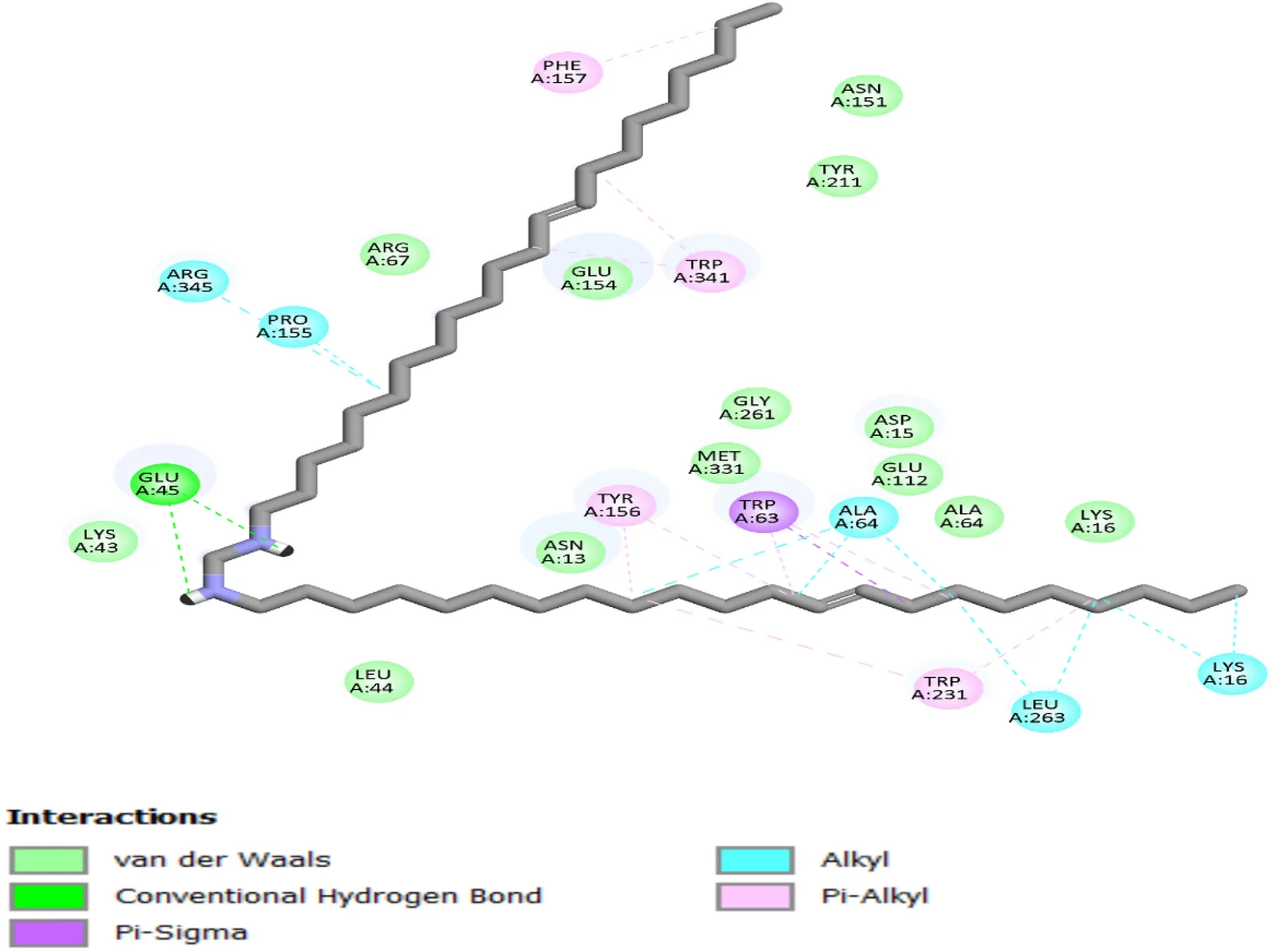
2D interaction diagram of the N, N-Methylene-Bis (14-Docosene) ligand docked into the active site of the HPV16 E6 protein (PDB ID: 7UAJ).

**Fig. 19:**
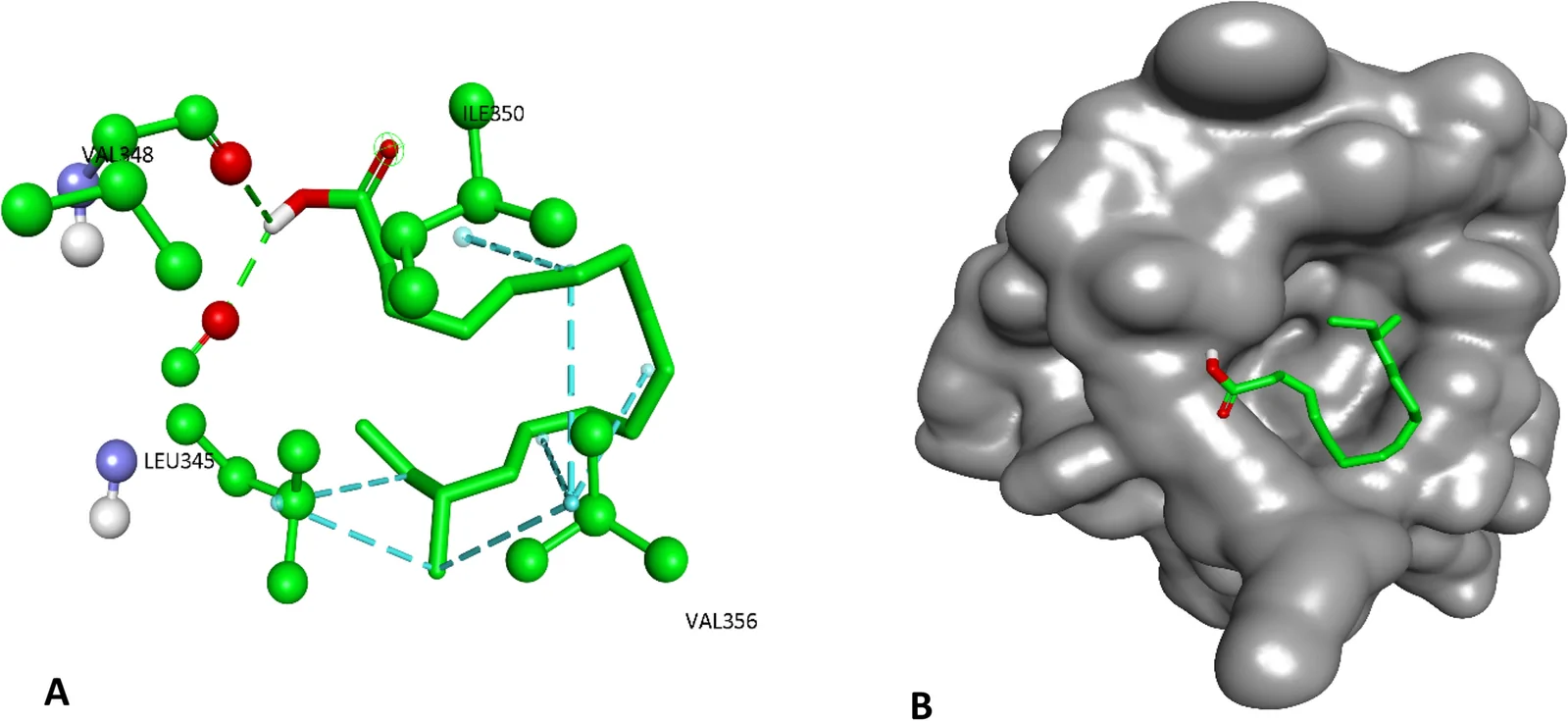
3D visualization of the molecular docking interactions between 14-Methylhexadecanoic acid and the active site residues of HPV16 E2 (PDB: 2Q79). (**A**) Detailed view of the binding interface, showing the ligand (green sticks) interacting with key hydrophobic residues (LEU345, VAL348, ILE350, VAL356). Hydrogen bonds and hydrophobic contacts are depicted as green and cyan dashed lines, respectively. (**B**) Surface representation of the binding pocket in the same orientation. The grey surface reveals the deep hydrophobic cavity that exhibits high steric complementarity with the ligand, confirming the tight fit of the methylated fatty acid chain.

**Fig. 20:**
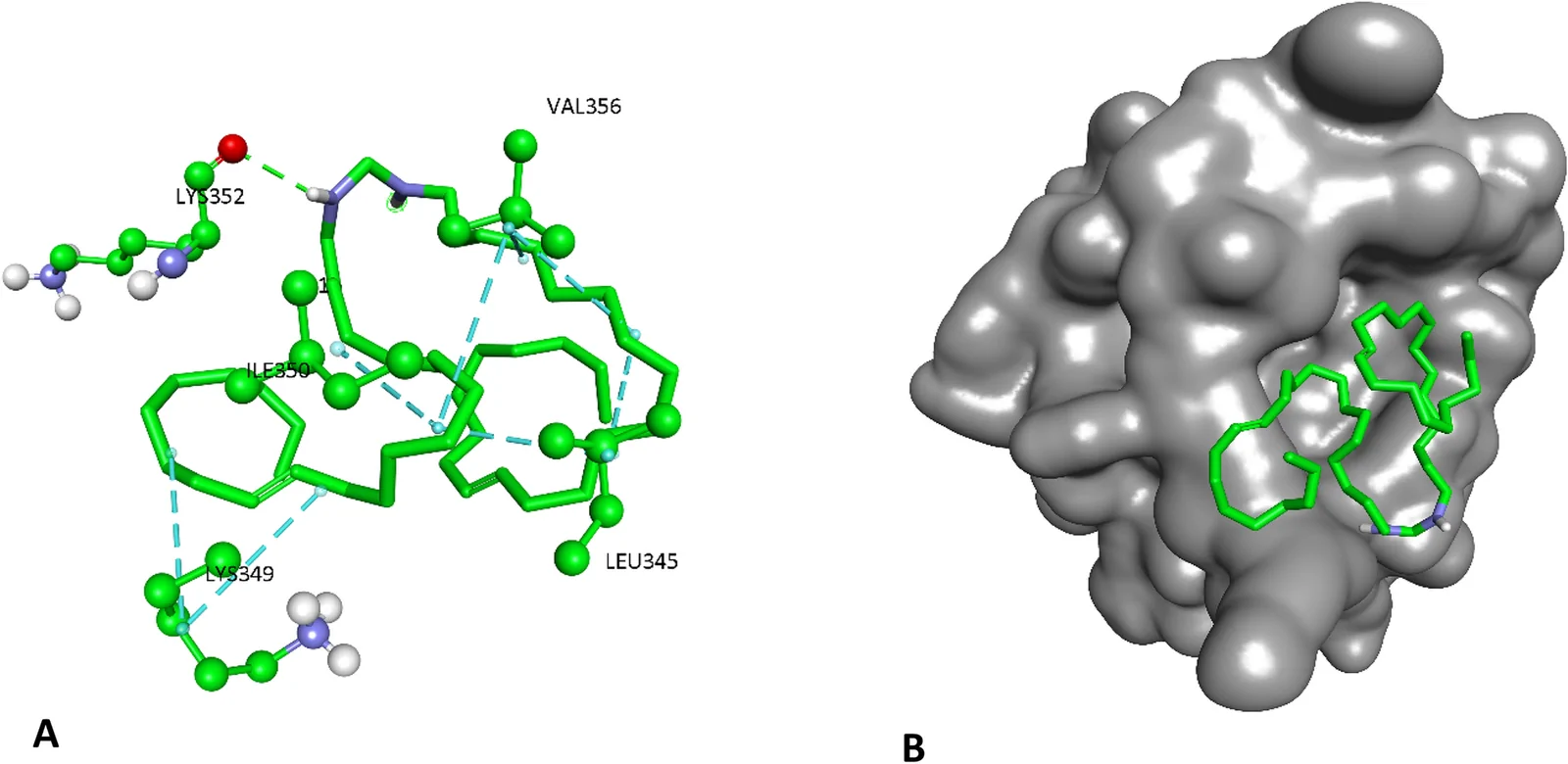
Molecular docking profile and binding determinants of N, N-Methylene-Bis (14-Docosene) within the HPV16 E2 active pocket (PDB: 2Q79). (**A**) Detailed 3D interaction map. The ligand (green sticks) is anchored by a key hydrogen bond with **LYS352** (green dashed line). The long hydrocarbon chain is further stabilized by hydrophobic interactions with **LYS349**, **LEU345**, **ILE350**, and **VAL356** (cyan dashed lines). (**B**) Surface representation of the binding site. The grey surface illustrates the accommodation of the ligand's elongated chain within the hydrophobic groove, highlighting the shape complementarity between Docosene and the viral protein interface.

**Fig. 21:**
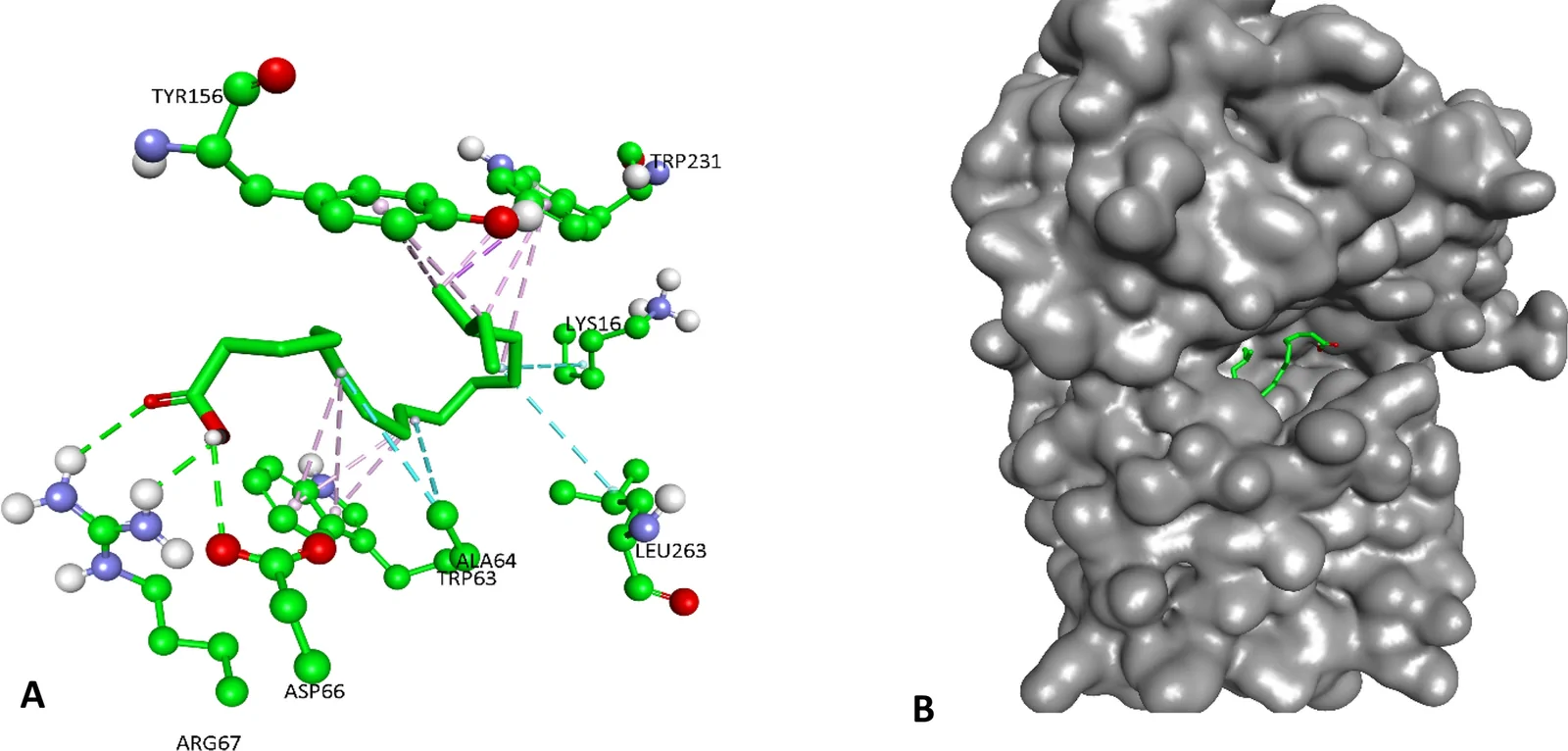
Molecular docking analysis of 14-Methylhexadecanoic acid within the active site of the HPV16 E6 protein (PDB ID: 7UAJ). (**A**) 3D view detailing the putative binding interactions between the ligand and key active site residues. Green dashed lines indicate conventional hydrogen bonds (with ARG67 and ASP66), while purple lines represent hydrophobic interactions involving aromatic residues such as TYR156 and TRP231. (**B**) Surface representation of the protein, illustrating the geometric accommodation and deep embedding of the ligand within the binding pocket.

**Fig. 22:**
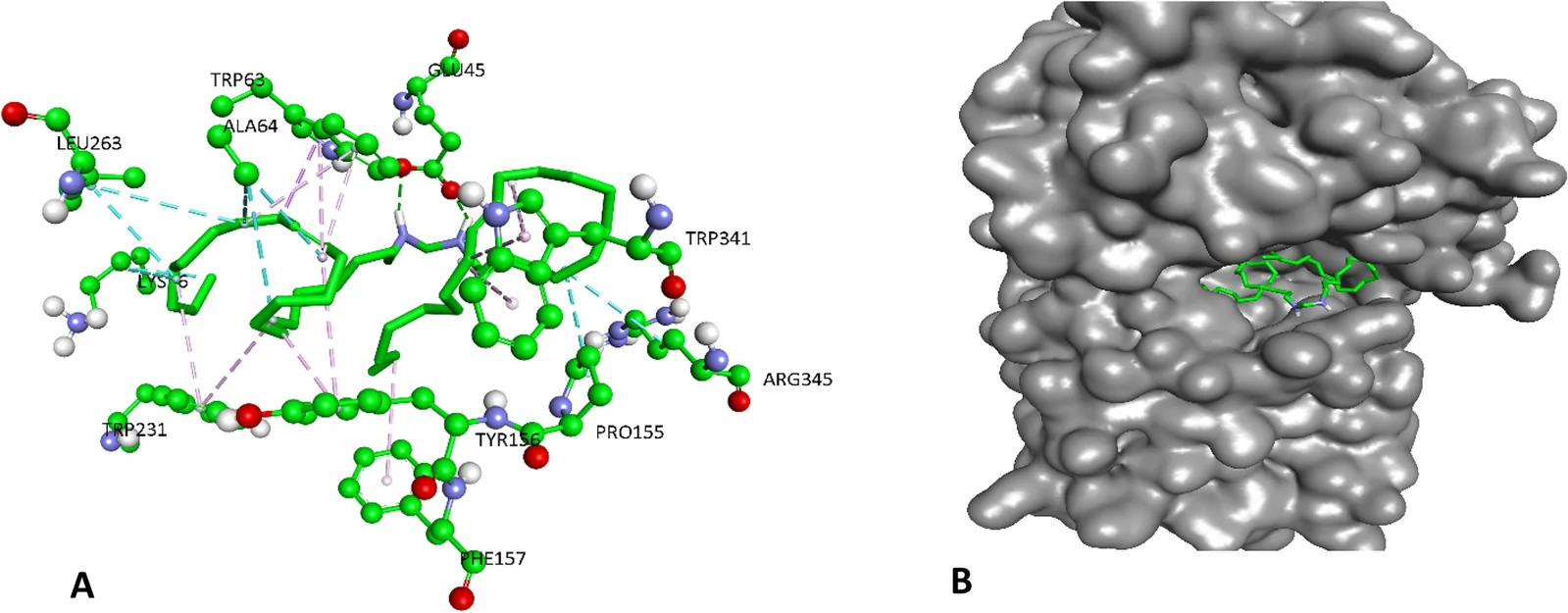
Molecular docking analysis of N, N-Methylene-Bis (14-Docosene) within the active site of the HPV16 E6 protein (PDB ID: 7UAJ). (**A**) 3D visualization of the ligand-receptor interaction network. Key amino acid residues involved in the binding are labeled. Dashed lines represent intermolecular interactions: cyan lines indicate hydrogen bonds, while pink/purple lines represent hydrophobic interactions (e.g., involving TYR156, TRP63). (**B**) Surface representation of the protein, illustrating the conformational fit and deep embedding of the ligand within the binding pocket.

Comparative induced-fit docking simulations were executed for 14-methylhexadecanoic acid and N, N-methylene-bis(14-docosene) against the HPV16 E6 oncoprotein (PDB: 7UAJ) and the HPV16 E2 regulatory protein (PDB: 2Q79); predicted binding affinities are detailed in Table 11 and Figs. 15–22. The predicted binding affinities reveal a target-specific dependency on ligand chain length and saturation, underscoring a Structure–Activity Relationship (SAR) in which steric volume and conformational flexibility dictate binding efficacy, consistent with established fatty acid–protein interaction principles.

Structural analysis revealed that hexadecanoic acid acts as an optimal scaffold for the E6 oncoprotein: its aliphatic tail engages in a dense network of van der Waals interactions, maximizing contact surface area and achieving geometric complementarity within the binding pocket (Figs. 17, 21). In contrast, 1,14-docosene displayed reduced affinity due to an “entropy factor” — its extended chain introduces a high degree of conformational flexibility that imposes a substantial entropic penalty upon binding and given the intrinsic conformational disorder of the HPV16 E6 oncoprotein, the energetic cost of stabilizing both a flexible ligand and a disordered protein region outweigh the enthalpic gains. This balance between surface-area coverage and entropic cost explains the superior affinity of the shorter derivative relative to the docosene derivative (Figs. 18, 22).

Conversely, 14-methylhexadecanoic acid acts as an optimal scaffold for the E2 regulatory protein (Figs. 15, 19); the bulky 14-docosene molecule instead requires an energetically unfavorable “coiled” conformation to fit into the E2 active site (Figs. 16, 20). Unlike E6, which is limited primarily by entropy, the E2 pocket appears to impose strict steric constraints on ligand volume.

Against the HPV18 E6 domain (6SJV), both phytochemicals showed reduced binding affinity relative to their HPV16 E6 (7UAJ) interactions: N,N-methylene-bis(1,14-docosene) yielded − 5.59 kcal/mol (vs. − 7.4 kcal/mol against 7UAJ), and 14-methylhexadecanoic acid yielded − 4.88 kcal/mol (vs. − 7.1 kcal/mol against 7UAJ) (Table 11). This trend parallels the chain-length and steric-bulk dependency established for the HPV16 targets, suggesting that the HPV18 E6 binding pocket is comparatively less accommodating of extended aliphatic substituents.

## Discussion

### Novelty and originality

Herbal remedies documented in developing countries with long-established medicinal traditions, such as Egypt and China, constitute a rich pharmacopeia that has been utilized for the treatment of numerous ailments for centuries, and increasing scientific attention has been directed toward the natural products that form the foundation of these traditional systems. The collection of wild medicinal plants from the North Coast Road, Egypt (Table 1), provides valuable insight into the antioxidant potential of flora native to this distinctive geographical region, where environmental factors such as soil salinity and climatic conditions play a crucial role in the biosynthesis of biologically active compounds. The markedly arid environment of Wadi El-Natrun, in contrast to the more Mediterranean-influenced coastal zone of the North Coast, represents distinct biotic and abiotic conditions that profoundly influence plant phytochemical profiles. These findings are consistent with those reported by^48^, who demonstrated that the phytochemical composition and biological activities of selected wild medicinal plants from Egypt's coastal and inland desert regions were significantly higher compared with those of non-wild plants.

The principal novel contribution of this work, relative to previous phytochemical and biological studies on *N. glauca*^19–24^, is the explicit quantification of synergistic and antagonistic interactions between the crude extract and its isolated pure compounds using the Combination Index method, applied consistently across both antioxidant and anticancer endpoints. This analysis reveals that the crude extract's biological potency is not fully explained by either isolated compound alone but depends on a synergistic interaction with Compound A (CI = 0.53 antioxidant; CI = 0.139–0.158 anticancer), a relationship not previously reported for this species. The linkage of this synergy to a specific mechanistic readout (Bax/Bcl-2 modulation and MDA elevation) and to a computational docking assessment of the species' predominant alkaloid against HPV oncoprotein targets further distinguishes the present integrated approach from prior, single-endpoint studies of *N. glauca*.

### Phytochemical profile of the screened wild plants

The occurrence of secondary metabolites in wild plants is of considerable importance to humankind, as these compounds exhibit a broad spectrum of therapeutic activities. Nevertheless, such metabolites are primarily produced as part of the plants' defense mechanisms against herbivores and various environmental stresses^49^.

These results are in agreement with previous data obtained by^50^, who reported that *N. tabacum* extract has higher amounts of phenols, which could indicate that *N. tabacum* extracts might contain a large number of phenolic compounds that exert antioxidant and other biological properties and are considered to be antifungal, antibacterial, antioxidant, and antitumor, as recorded by^51^ and^52^. These findings are also consistent with the well-known role of nicotine as a major alkaloid generated via ornithine metabolism^53^.

The phenolic content obtained is in agreement with^54^ (TPC values reaching 14.41 g/100 g in methanolic extracts). The flavonoid findings are consistent with^55^, which demonstrated significant pharmacological efficacy at similar concentration levels; the correlation between the high phenolic content and the significant flavonoid presence (2.14 g/100 g) strongly suggests that flavonoids are a major contributor to the total antioxidant capacity of *C. bonariensis*.

The substantial alkaloid accumulation observed in *N. glauca* is plausibly ascribed to the dominance of anabasine, the principal piperidine alkaloid of this species, known to be upregulated under environmental stress^56^; the elevated alkaloid levels observed here corroborate the potent toxicological and pharmacological character associated with *N. glauca*^24^. These findings are consistent with those reported by^16^, who demonstrated that four major alkaloids — nicotine, nornicotine, anabasine, and anatabine — accumulate in Nicotiana species and collectively account for the majority of total alkaloid content; in the present study, *N. glauca* exhibited the highest alkaloid content among the examined plants, confirming its well-documented role as a rich natural source of alkaloids, in agreement with previous reports^17,18^.

### Antioxidant activity of wild plants' maceration extracts

A strong positive correlation was observed among the four antioxidant assays (ABTS vs. KMnO₄, r = 0.92; ABTS vs. DCPIP, r = 0.93; DPPH vs. KMnO₄, r = 0.90), indicating that the assays consistently capture comparable electron-donating and radical-scavenging mechanisms. These results are broadly consistent with previous reports on N. glauca^19^ and related desert species^57,58^, which similarly linked phenolic and flavonoid content to strong DPPH/ABTS activity. However, as shown in the correlation analysis (Table 4), this association did not reach statistical significance for any single compound class in the present dataset, suggesting a combined rather than single-class contribution to the observed activity.

Numerous epidemiological studies have demonstrated that the consumption or utilization of wild plants rich in phenolic and flavonoid compounds with potent antioxidant properties is associated with a reduced incidence of cardiovascular diseases, cancer, diabetes, and neurodegenerative disorders^59^. In line with this evidence, the present findings are consistent with previous reports by^19^, who showed that the methanolic extract of N. glauca contained a high concentration of phenolic compounds (111 µg gallic acid equivalents/g extract) and exhibited strong antioxidant activity, as reflected by 94.80% DPPH and 97.57% ABTS inhibition, in addition to notable anti-inflammatory effects (81.93% inhibition). Furthermore^20^, reported that the stem extract of *N. tabacum* is enriched with antioxidant enzymes, such as glutathione transferase and superoxide dismutase, which further enhance its antioxidant potential.

Similarly^57^, reported that ethanolic extracts of *A. monosperma* and *Limbarda crithmoides* (L.) Dumort. exhibited high DPPH radical scavenging activity, with values comparable to or exceeding those of ascorbic acid. In addition^58^, demonstrated a strong positive correlation (R^2^ = 0.91) between free radical scavenging activity and phenolic content in ethyl acetate and acetone extracts of *T. hirsuta*.

In the present study, the aqueous ethanolic extract of *N. glauca* exhibited the highest antioxidant activity among the tested plants, which can be largely attributed to its high content of bioactive compounds, particularly phenolics, flavonoids, and alkaloids (Table 3). These findings agree with previous studies^44,60^, which reported a strong correlation between antioxidant activity (as determined by DPPH and ABTS assays) and phenolic compound concentrations in various plant species and algae.

It should be emphasized that DPPH, ABTS, KMnO₄ and DCPIP are chemical, cell-free antioxidant assays; the present findings characterize the chemical radical-scavenging and reducing capacity of *N. glauca* extracts and should not be interpreted as evidence of cellular or in vivo antioxidant efficacy, which remains to be established.

### Fractionation, sub-fractionation and combination index for antioxidant activity

Based on the TLC bioautography results, *A. monosperma*, *T. hirsuta*, and *N. glauca* exhibited particularly high levels of antioxidant compounds, with *N. glauca* displaying the most abundant antioxidant constituents among the studied plants, consistent with the fraction-level results below.

Fractions 8–11 being identified as the most potent antioxidant fractions of *N. glauca* suggests enrichment of bioactive compounds with strong free radical scavenging potential, with implications for their potential use in food, pharmaceutical, and cosmetic applications. Based on the antioxidant activity results of the crude extracts and their semi-purified fractions (Tables 5 and 6), it can be concluded that fractionation generally led to a reduction in activity, with most fractions exhibiting lower antioxidant capacity than the corresponding crude extract. This suggests that the strong activity observed in the crude extract may result from the combined or synergistic action of bioactive compounds distributed across one or more of the sixteen fractions. These findings are consistent with previous reports^44,61^, which demonstrated that crude methanolic extracts of Arthrospira sp. exhibited higher antioxidant activity than isolated pure components; in these cases, constituents such as flavonoids and phenolic compounds acted collectively, producing greater antioxidant effects than any single fraction or compound alone.

The presence of multiple antioxidants in the crude extract may generate synergistic interactions that enhance overall activity. For example, the observation that Compound A — the primary active component — was isolated from subfraction 1, which showed comparatively low total antioxidant activity, suggests that its full potency requires the presence of other co-factors or complementary compounds within the crude extract. This highlights the importance of cooperative interactions among extract constituents and reinforces the significance of Combination Index values in evaluating the synergistic relationships necessary to achieve optimal therapeutic effects.

These results are consistent with those reported by^62^, who demonstrated that antioxidant activity is closely related to the chemical structure of the compounds, particularly the number and type of functional groups, such as hydroxyl (–OH) or amine (–NH₂) groups; not only the number but also the position of these active groups significantly influences the antioxidant potential. Furthermore, the observed antioxidant activity in the present study was directly proportional to the concentration of phenolic compounds in the extracts (Table 3). The GC/MS/MS findings for Fraction 11 are in agreement with previous reports^23^, where GC–MS analysis of *N. glauca *indicated anabasine as the predominant compound, while nicotine was not detected; this contrasts with the markedly different composition (rich in oxygenated sesquiterpenes such as β-bisabolol) reported for the volatile oil obtained by hydrodistillation from N. glauca leaves, where anabasine was absent. Collectively, these results indicate that Fraction 11 contains a concentrated mixture of nitrogenous alkaloids, particularly anabasine and nornicotine, which may contribute to its pronounced antioxidant and cytotoxic activities.

The compound isolated from subfraction 7, 14-methylhexadecanoic acid, has been reported previously in *N. glauca* and other medicinal plants^44^, and its antioxidant potential has been confirmed in earlier studies^45–47^.

The pronounced synergistic effect between the crude extract and Compound A (CI = 0.53) indicates that the antioxidant activity of the mixture surpasses the sum of the activities of the individual components; such synergistic interactions are increasingly recognized in studies of plant-derived antioxidants, where complex mixtures of phytochemicals can act cooperatively to enhance radical scavenging and other antioxidant mechanisms^63^. In contrast, the strong antagonistic effect with Compound B (CI = 28.8) suggests that Compound B substantially inhibits the antioxidant activity of the crude extract, potentially through competitive inhibition of active antioxidant components or induction of pro-oxidant effects under the tested conditions^64^. These findings underscore the importance of understanding interactions among components in complex natural extracts and the need for careful evaluation of potential negative interactions when isolating and combining natural compounds for antioxidant applications^65^.

### Cytotoxicity and combination index for anticancer activity

This cytotoxicity result aligns with recent research highlighting the anticancer potential of *N. glauca* extracts against various cancer cell lines, including MCF7 breast cancer cells^66^. The strong activity of the crude extract likely results from the synergistic action of its multiple bioactive compounds, a phenomenon increasingly recognized in plant-based anticancer research^67^. The reduced potency of Fraction 11 compared to the crude extract suggests that the fractionation process might have disrupted the synergistic interactions between compounds or diluted key active components.

Benchmarking directly against doxorubicin (IC₅₀ = 0.88 ± 0.02 µg/mL in HeLa; 0.91 ± 0.04 µg/mL in HepG2) confirms, as would be expected when comparing a purified clinical chemotherapeutic with a crude plant extract, that *N. glauca* and its isolated compounds are markedly less potent in absolute terms. This benchmark does not diminish the relevance of the present findings; rather, it appropriately positions the crude extract's activity as that of an early-stage natural-product lead rather than a drug-ready candidate and confirms that the assay system used here is pharmacologically sensitive and valid.

The isolated Compound A showing the least cytotoxic activity across all tested cell lines suggests that this compound alone does not account for the significant cytotoxic effects observed in the crude extract and Fraction 11 and is consistent with a possible synergistic contribution among multiple constituents within the crude extract (Table 9). The observed high cell viability at lower concentrations for the HepG2 cell line across all tested samples is a crucial aspect in anticancer drug development, as selective action can minimize off-target effects on healthy cells; further research to identify the compounds responsible for this selective action is warranted.

The enhanced potency of the crude N. glauca extract against HeLa and HepG2 cell lines compared to Fraction 11 and Compound A may be attributed to an auto-synergistic effect among the various components within the crude extract (Table 8), aligning with the concept that complex mixtures in crude extracts can exhibit greater biological activity due to the cooperative interactions of their constituents. Furthermore, the presence of minor secondary metabolites in the crude extract could potentiate the activity of the main compounds and enhance their stability against enzymatic degradation by tumour cells, a mechanism observed in antioxidant studies of plant extracts^68^.

The synergistic interaction between the crude extract and Pure Compound A (CI = 0.139 in HeLa; CI = 0.158 in HepG2) indicates that the combined effect is greater than the sum of their individual effects, suggesting potential for enhanced anticancer efficacy when used in combination^68^. Conversely, the antagonistic interaction with Pure Compound B (CI = 2.2) implies that these two components interfere with each other's anticancer activity in HeLa cells. These results agree with previous research by^21^, who reported significant anti-cancer activity of *N. glauca* water extracts against MCF-7 and HeLa cell lines, with notable growth inhibition of HeLa cells at various concentrations. Our findings extend this by demonstrating the potential for synergistic interactions between the crude extract and a specific isolated compound, highlighting the complexity and potential of *N. glauca* as a source of anticancer agents. Understanding these synergistic relationships is crucial for developing more effective and targeted anticancer therapies.

As clarified above, Compound A is not the most potent cytotoxic agent in isolation — its stand-alone IC₅₀ values in HeLa and HepG2 cells are consistently higher (weaker) than those of the crude extract (Fig. 10, Table 8). Rather, Compound A's significance lies in its capacity to potentiate the crude extract's activity when combined, as reflected in the strongly synergistic CI values obtained for both antioxidant and anticancer combinations. Compound A is therefore best understood as a key synergy partner within the crude extract's phytochemical matrix, rather than as its principal active molecule in an absolute, stand-alone sense; the crude extract's superior activity likely reflects the cooperative contribution of multiple co-occurring constituents, consistent with the antioxidant fractionation data (Tables 6 and 8).

### Gene expression and oxidative stress mechanism

In untreated control cells, the Bax/Bcl-2 ratio of approximately 1.0 indicated a balanced apoptotic threshold. Compound A treatment was associated with a substantial shift in this balance, consistent with the initiation of apoptosis, or programmed cell death. This observation is consistent with recent findings highlighting the critical role of the Bax/Bcl-2 ratio as a rheostat determining cellular fate in response to anticancer agents^22^. An elevated Bax/Bcl-2 ratio is widely recognized as a key trigger of the mitochondrial pathway of apoptosis in various cancer cell types^69,70^.

Consistent with the pro-apoptotic gene expression profile, our findings are consistent with a possible mitochondrial-centric mechanism of action for Compound A. The observed increase in Bax and decrease in Bcl-2 may plausibly disrupt the mitochondrial outer membrane potential, a crucial early event in apoptosis, potentially leading to the release of pro-apoptotic factors such as cytochrome c and subsequent activation of the caspase cascade, which would ultimately result in cell death^71^. The increased MDA levels observed after Compound A treatment further indicate increased oxidative stress, which can damage mitochondrial components, including DNA and membranes, exacerbating the apoptotic process and hindering cancer cell survival^72,73^. This substantial elevation in MDA is consistent with, though not direct proof of, Compound A being associated with significant oxidative stress in HeLa cells, contributing to cellular damage and potentially playing a key role in its anticancer efficacy. Recent studies have increasingly highlighted the role of oxidative stress induction as a viable strategy for cancer therapy by overwhelming the antioxidant defense mechanisms of cancer cells^74^. The potent cytotoxic effect of Compound A, as indicated by its low IC₅₀ value, further underscores its potential as an anticancer agent.

The mitochondrial and caspase-cascade steps described above were not directly measured in this study and are proposed based on the Bax/Bcl-2 and MDA data; direct confirmation (Annexin V/PI, caspase-3/7 activity, PARP cleavage, or TUNEL assays) is required before apoptosis induction can be considered established. Likewise, MDA is a downstream lipid-peroxidation biomarker and not a direct measure of intracellular reactive oxygen species (ROS); ROS-specific probes (e.g., DCFDA fluorescence or flow-cytometric ROS assays) are needed to confirm oxidative stress directly.

### Molecular docking of anabasine and SAR analysis (independent computational screen, decoupled from cytotoxicity mechanism)

We reiterate that this docking analysis is a fully independent computational screen, decoupled from the cytotoxicity mechanism discussed above. Because HeLa cells are HPV18-positive rather than HPV16-positive, the docking analysis against the HPV16 E2 (2Q79) and E6 (7UAJ) oncoproteins does not, and is not intended to, establish a direct mechanistic relationship with the cytotoxicity observed in the HeLa or HepG2 assays reported above. It is presented purely as an exploratory assessment of anabasine's potential activity against high-risk HPV oncoproteins as a structural class, motivated by anabasine's identity as the predominant alkaloid in the most bioactive fraction (Fraction 11) rather than by any cell-line-specific rationale; docking against HPV18-specific oncoprotein structures is identified as a priority for future work (see Conclusion).

The binding energy obtained for 2Q79 (− 7.4638 kcal/mol) is consistent with established literature, where similar ligands, such as nicotine derivatives targeting related protein families, have demonstrated binding energies within the − 6 to − 8 kcal/mol range^75,76^. The hydrogen bonds formed between anabasine and residues Lys349 and Ile350 are pivotal for anchoring the ligand and contribute significantly to the specificity and stability of the ligand–protein complex, a phenomenon well-supported by previous docking studies^77^; the hydrophobic interaction with Leu345 complements this hydrogen bonding network, consistent with existing research underscoring the importance of hydrophobic contacts in stabilizing alkaloid–protein interactions^77^.

To extend this comparative assessment across HPV genotypes, anabasine was additionally docked against the HPV18 E6 domain (6SJV), under the same AutoDock Vina protocol and exhaustiveness setting (56) used for 2Q79 and 7UAJ, yielding a binding affinity of − 5.64 kcal/mol — a moderately weaker interaction relative to the HPV16 targets (2Q79: − 7.4638 kcal/mol; 7UAJ: − 7.2349 kcal/mol). This reduced affinity toward HPV18 E6 may reflect structural divergence in the ligand-binding pocket between the two high-risk genotypes, despite their shared oncogenic mechanism via E6AP-mediated p53 degradation. Nonetheless, the consistent negative binding energy across all three targets supports anabasine's broad, if genotype-dependent, potential to engage high-risk HPV E6/E2 proteins as a structural class.

These findings provide computational support consistent with the promising therapeutic potential of *N. glauca* extract and its active compounds as antioxidant and anticancer agents, highlighting their potential molecular mechanisms of action and opening new avenues for the development of effective natural cancer therapies. The detailed interaction profiles illustrated in the 2D and 3D interaction diagrams (Figs. 11–14) are computational predictions that support the potential of anabasine as a candidate for drug development targeting these proteins, pending experimental validation.

These docking results should be interpreted as computational predictions of binding affinity and pose, providing hypothesis-generating support for anabasine's potential interaction with HPV16 E2 and E6 oncoproteins; they do not constitute direct experimental evidence of ligand–protein binding, and complementary biophysical or cell-based validation is required before mechanistic conclusions can be drawn.

The predicted binding affinities for the SAR analysis reveal a target-specific dependency on ligand chain length and saturation, underscoring a critical Structure–Activity Relationship (SAR) where steric volume and conformational flexibility dictate binding efficacy, consistent with established fatty acid-protein interaction principles^78^. Structural analysis reveals that hexadecanoic acid acts as an optimal scaffold for the E6 oncoprotein, with its aliphatic tail engaging in a dense network of van der Waals interactions that maximize contact surface area; this aligns with findings identifying the hydrophobic properties of E6 oncoproteins as critical for their interaction with cellular partners^79^ (Figs. 17, 21). In contrast, 1,14-docosene displayed reduced affinity due to the “entropy factor”: its extended chain introduces a high degree of conformational flexibility, imposing a substantial entropic penalty upon binding^80^, and given the intrinsic conformational disorder of the HPV16 E6 oncoprotein^81^, the energetic cost of stabilizing both a flexible ligand and a disordered protein region outweighs the enthalpic gains — a balance between surface area coverage and entropic cost that explains the superior affinity of the shorter derivative compared to the docosene derivative (Figs. 18, 22).

Conversely, 14-methylhexadecanoic acid acts as an optimal scaffold for the E2 regulatory protein (Figs. 15, 19); for the bulky 1,14-docosene molecule, fitting into the E2 active site requires the adoption of an energetically unfavorable “coiled” conformation (Figs. 16, 20). This suggests that, unlike E6, which is limited by entropy, the E2 pocket imposes strict steric constraints on ligand volume.

A similar genotype-comparative trend was observed for the two phytochemicals: N,N-Methylene-Bis(14-Docosene) and 14-Methylhexadecanoic acid exhibited binding affinities of − 5.59 and − 4.88 kcal/mol, respectively, against the HPV18 E6 domain (6SJV) — both weaker than their corresponding interactions with the HPV16 E6 oncoprotein (7UAJ: − 7.4 and − 7.1 kcal/mol, respectively). This pattern is consistent with the SAR trend established for HPV16 E6, in which chain length and steric bulk modulate binding efficacy, and further suggests that the HPV18 E6 binding pocket may be comparatively less accommodating of extended or bulky aliphatic substituents than its HPV16 counterpart. Taken together, these cross-genotype comparisons indicate that while the docked ligands retain measurable affinity for HPV18 E6, HPV16-specific targets remain the more favourable docking partners among the systems examined here.

### Limitations of the study

This study has several limitations that should be considered when interpreting its findings. First, apoptosis was inferred from Bax/Bcl-2 gene expression alone; while the observed shift in the Bax/Bcl-2 ratio is consistent with a pro-apoptotic effect, direct confirmation using Annexin V/PI flow cytometry, caspase-3/7 activity assays, PARP cleavage, or TUNEL staining was not performed and is needed to substantiate this mechanism. Second, oxidative stress was assessed only indirectly via the lipid-peroxidation biomarker MDA; direct intracellular ROS measurement (e.g., DCFDA fluorescence) would provide more definitive evidence. Third, the molecular docking results represent computational binding predictions only, generated against HPV16 oncoprotein structures as an exploratory, hypothesis-generating screen independent of the HPV18-positive HeLa cell line used in the cytotoxicity assays, and require experimental validation (e.g., binding assays or co-crystallization) before the proposed anabasine–HPV oncoprotein interactions can be considered confirmed. Although a preliminary docking analysis against the HPV18 E6 domain (6SJV) was included in this study, it was limited to a single representative crystal structure; broader analyses incorporating multiple HPV18 E6 structures or strain variants, together with complementary experimental validation (e.g., E6 knockdown or p53 restoration assays), are planned as part of future work to more comprehensively assess anabasine's genotype-specific anti-HPV18 potential. Finally, all biological assays were conducted in vitro; in vivo efficacy, pharmacokinetics, and toxicity remain to be evaluated.

### Safety considerations regarding *N. glauca* alkaloids

*N. glauca* accumulates anabasine as its predominant alkaloid, a compound with well-documented toxicity, including a reported case of fatal human poisoning following ingestion of *N. glauca* leaves^24^. This narrow margin between the biologically active and toxic doses of anabasine represents an important consideration for any future translational development of *N. glauca*-derived extracts or fractions. Selectivity-index studies comparing cytotoxicity in cancer versus normal cell lines (as reported here for Compound B, Table 9) should be extended to the crude extract, Compound A, and anabasine-enriched fractions before in vivo work is undertaken. Where possible, future work should also evaluate whether the non-alkaloid constituents of the extract (e.g., Compound A and B) can reproduce the observed synergistic antioxidant/anticancer activity at alkaloid-sparing concentrations, potentially offering a safer path toward therapeutic application.

**Fig. 23:**
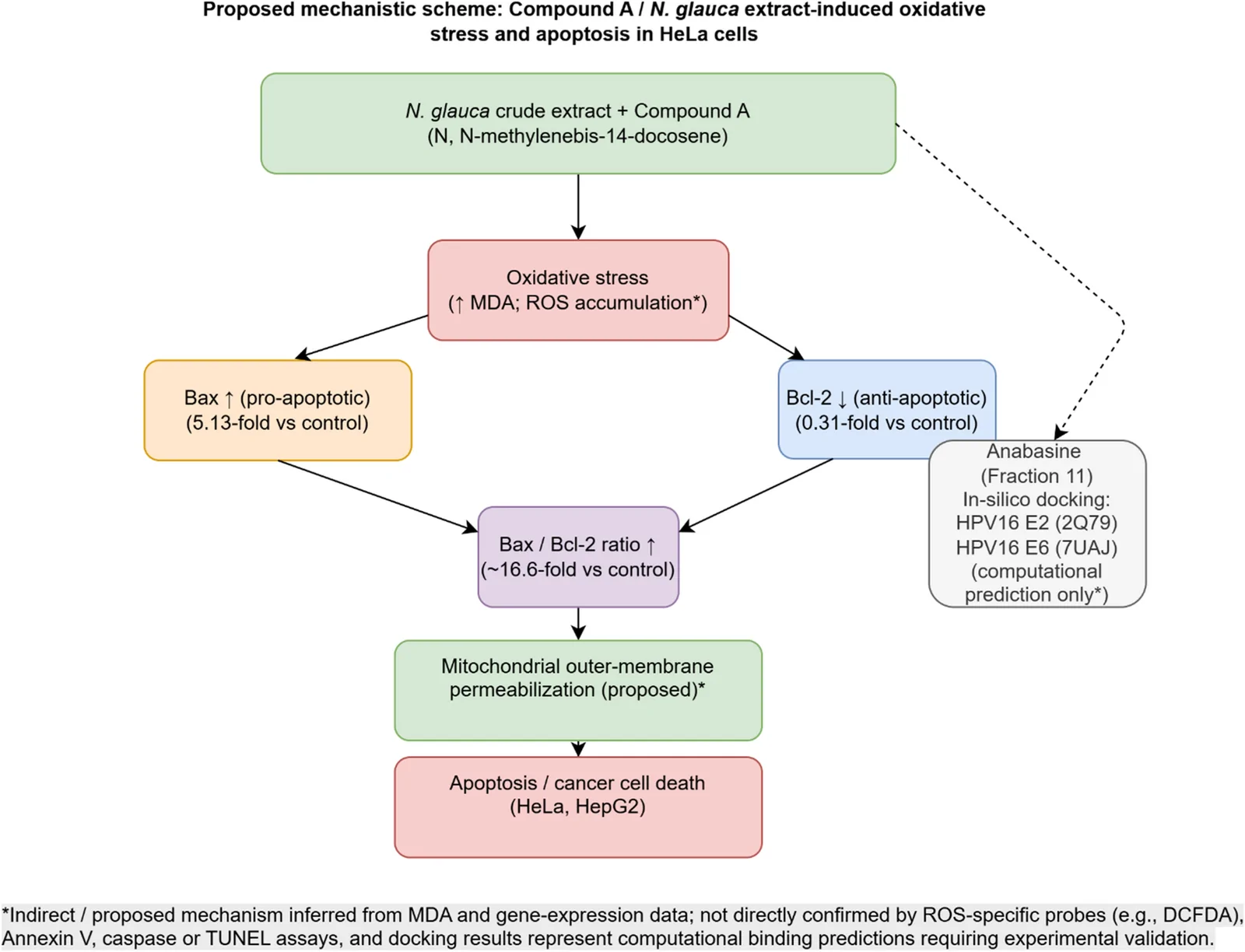
Proposed mechanistic scheme summarizing the effect of the *N. glauca* crude extract and Compound A on HeLa cells, distinguishing directly supported steps (solid arrows) from proposed/computationally predicted links (dashed arrows).

Figure 23 proposed mechanistic scheme summarizing the effect of the *N. glauca* crude extract and Compound A on HeLa cells. Solid arrows indicate steps directly supported by the gene-expression (Bax, Bcl-2) and MDA data reported in this study (crude extract/Compound A → oxidative stress [↑MDA] → Bax↑/Bcl-2↓ → increased Bax/Bcl-2 ratio); dashed arrows and asterisked steps indicate proposed or computationally predicted links (mitochondrial outer-membrane permeabilization, apoptosis/cancer cell death in HeLa and HepG2, and anabasine–HPV oncoprotein docking against 2Q79/7UAJ) that were not directly measured in this study and require further experimental confirmation.

## Conclusion

The current study characterizes the wild plant *N. glauca*, selected from a ten-species screening on the basis of its high extraction yield and phytochemical richness, as a source of bioactive antioxidant and cytotoxic metabolites. Its principal isolated compound, N,N-methylene bis(14-docosene) (Compound A), was not the most potent cytotoxic or antioxidant agent in isolation, but acted as a key synergy partner, markedly potentiating the crude extract's antioxidant and anticancer activity (CI = 0.53 and 0.139–0.158, respectively), whereas 14-methylhexadecanoic acid (Compound B) antagonized this activity. Treatment of HeLa cells with Compound A was associated with an increased Bax/Bcl-2 ratio and elevated MDA levels, findings consistent with, though not direct proof of, apoptosis induction and oxidative stress. Molecular docking suggested that anabasine, the extract's predominant alkaloid, binds with favorable predicted affinity to HPV16 E2 and E6 oncoproteins, a computational finding requiring experimental confirmation.

Building on these findings, future work should prioritize: (i) direct confirmation of apoptosis (Annexin V/PI, caspase-3/7, TUNEL) and of oxidative stress (DCFDA-based ROS quantification); (ii) experimental validation of the anabasine–HPV oncoprotein docking predictions, ideally including docking against HPV18 oncoprotein structures to align directly with the HPV18-positive HeLa cell line used in this study; (iii) selectivity-index and in vivo toxicity evaluation of the crude extract, Compound A, and anabasine-enriched fractions, given the known toxicity of *N. glauca* alkaloids; and (iv) exploration of whether the extract's synergistic activity can be reproduced or enhanced using alkaloid-sparing compound combinations, as a step toward safer translational development.

## Supplementary Information

Supplementary Information 1.

Supplementary Information 2.

Supplementary Information 3.

## Data Availability

The datasets used and analyzed during the current study are available from the corresponding author on reasonable request.

## Abbreviations

ABTS: 2,2-azinobis (3-ethylbenzothiazoline-6-sulfonic acid)
CI: Combination index
DCPIP: Dichlorophenolindophenol
DPPH•: 2,2-diphenyl-1-picrylhydrazyl
EI: Electron ionization
FTIR: Fourier transfer infrared
GC/MS/MS: Gas chromatography/mass spectrum/ mass spectrum
NCDs: Noncommunicable diseases
NMR: Nuclear magnetic resonance
MDA: Malondialdehyde
TLC: Thin layer chromatography
